# Synthesis and Biological Activity of Triterpene–Coumarin Conjugates

**DOI:** 10.1021/acs.jnatprod.1c00128

**Published:** 2021-05-06

**Authors:** Karina Vega-Granados, Marta Medina-O’Donnell, Francisco Rivas, Fernando J. Reyes-Zurita, Antonio Martinez, Luis Alvarez de Cienfuegos, Jose A. Lupiañez, Andres Parra

**Affiliations:** † Departamento de Química Orgánica, Universidad de Granada, E-18071 Granada, Spain; ‡ Departamento de Bioquímica y Biología Molecular I, Universidad de Granada, E-18071 Granada, Spain

## Abstract

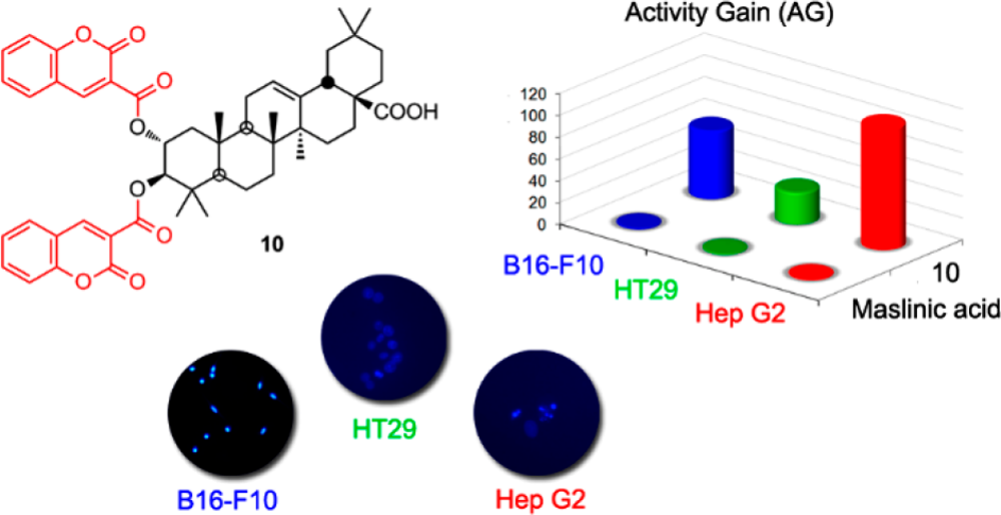

A set of 12 maslinic acid–coumarin conjugates was synthesized, with 9 being maslinic acid–diamine–coumarin conjugates at the C-28 carboxylic acid group of triterpene acid and the other three being maslinic acid–coumarin conjugates at C-2/C-3 and/or C-28 of the triterpene skeleton. The cytotoxic effects of these 12 triterpene conjugates were evaluated in three cancer cell lines (B16-F10, HT29, and Hep G2) and compared, respectively, with three nontumor cell lines from the same or similar tissue (HPF, IEC-18, and WRL68). The most potent cytotoxic results were achieved by a conjugate with two molecules of coumarin-3-carboxylic acid coupled through the C-2 and C-3 hydroxy groups of maslinic acid. This conjugate showed submicromolar IC_50_ values in two of the three cancer cell lines tested (0.6, 1.1, and 0.9 μM), being between 110- and 30-fold more effective than its corresponding precursor. Furthermore, this conjugate (**10**) showed percentages of cell viability for the three nontumor lines of 90%. Four maslinic acid–coumarin conjugates displayed apoptotic effects in the treated cells, with total apoptosis rates of between 40 and 85%, relative to the control. Almost all the compounds assayed caused cell-cycle arrest in all cancer cell lines, increasing the number of these cells in the G0/G1 phase.

Pentacyclic triterpenoids are natural products and, more specifically, secondary metabolites in plants and fungi that perform highly varied functions in living organisms, displaying antitumor,^1,2^ antiviral,^3,4^ antibacterial,^5,6^ and anti-inflammatory^7,8^ activity. For example, within the group of pentacyclic triterpenes with an oleanane skeleton, maslinic acid (2α,3β-dihydroxyolean-12-en-28-oic acid, MA, **I**)^9^ appears abundantly in industrial olive oil waste,^10^ attracting attention as a pharmacologically active product due to its varied biological properties, especially as an antitumor,^11,12^ antioxidant,^13^ and anti-inflammatory agent.^14,15^

In recent years, the synthesis of conjugates of pentacyclic triterpenes with other molecules has improved the biological properties of the parent compounds.^16^ Our research group has synthesized numerous conjugates of triterpenoid compounds, such as oleanolic and maslinic acids. These conjugates have been tested as potential antitumor agents, and some exhibit cytotoxicity with submicromolar values and reach a total apoptosis rate greater than 95%.^17−19^ In addition, these triterpenoid conjugates have also been assayed as antiviral agents against HIV-1 protease, and some show submicromolar values and an activity gain of 100 times greater than that of their natural precursors.^20^

Fluorescent labels are used commonly to visualize small molecules in cells as well as tissues. These fluorescent labels, coupled with a wide variety of compounds, have been used as detection probes in fluorescence microscopy.^21^ Pentacyclic triterpenes with fluorophore groups have not been employed widely as probes for biological studies, although a number of recent articles have examined this matter.^22−24^

In the present study, several commercial coumarins have been coupled to maslinic acid (MA, **I**), through the C-28 carboxylic acid group of this pentacyclic triterpene, using various primary diamine linkers with different chain lengths. Some of these commercial coumarins have been directly coupled to maslinic acid (**I**), through the C-2 and C-3 hydroxy groups of ring A and/or through the C-28 carboxylic acid group. The cytotoxic effects of these 12 maslinic acid–coumarin conjugates have been tested against three cancer cell lines (B16-F10, HT29, and Hep G2), and the results of the most active compounds were compared with three nontumor cell lines from the same or similar tissue (HPF, IEC-18, and WRL68). The best results were achieved by using a conjugate with two molecules of coumarin-3-carboxylic acid (C3CA), coupled through the C-2 and C-3 hydroxy groups of maslinic acid (**I**), which gave submicromolar IC_50_ concentrations on the cancer cell lines. Triterpene–coumarin conjugates are promising fluorescent probes for enabling the subcellular localization of drugs in cells or tissues.

## Results and Discussion

### Chemistry

**Scheme 1:**
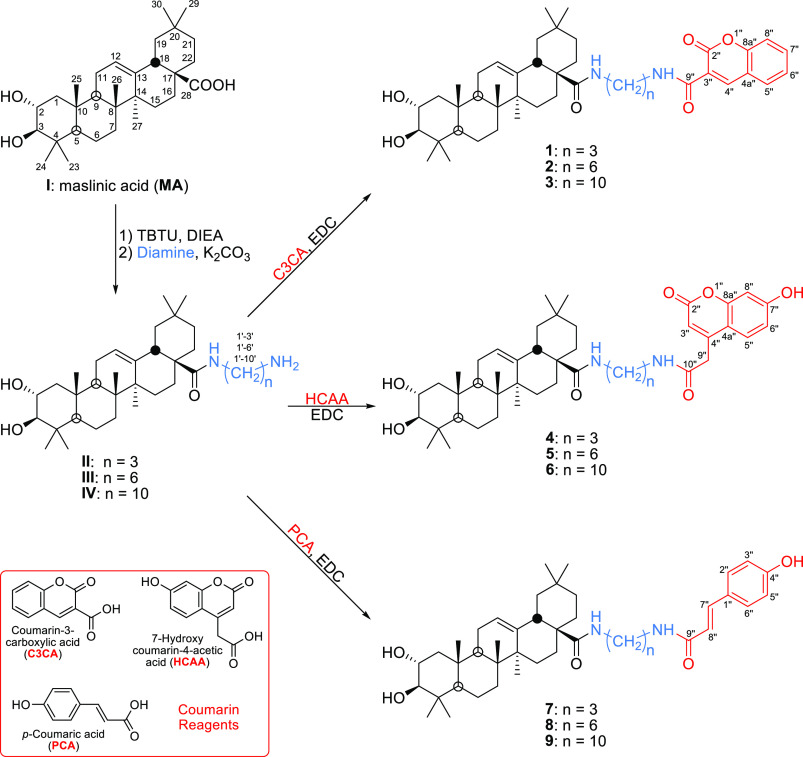
Synthesis of the MA–Diamine–Coumarin Conjugates **1–9**

**Scheme 2:**
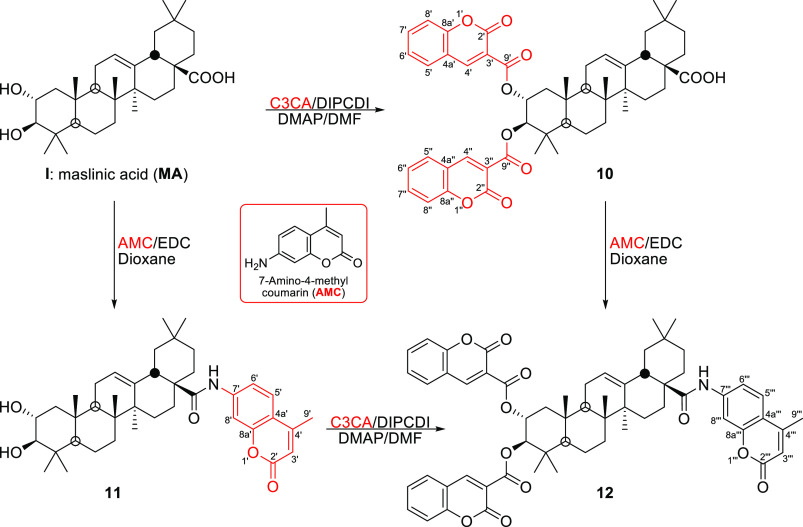
Synthesis of the MA–Coumarin Conjugates **10–12**

Maslinic acid (2α,3β-dihydroxyolean-12-en-28-oic acid, MA, **I**), isolated from olive oil industry waste, was used as the starting material to produce various triterpene–coumarin conjugates (Schemes 1 and 2). MA was obtained from its natural source, by means of an extraction procedure in a Soxhlet system, using ethyl acetate as solvent. Next, the MA was purified on a chromatographic column, using dichloromethane with increasing amounts of acetone as eluents.

Several C-28 conjugates of MA were synthesized in order to evaluate their biological properties. Nine MA–diamine–coumarin conjugates (**1**–**9**) were prepared by coupling three commercial coumarins to MA (**I**), through the C-28 carboxylic acid group of this triterpene (Scheme 1), using several primary diamines with different chain lengths (3, 6, or 10 methylene groups) as linkers, to evaluate their influence on the biological activities of these conjugates (**1**–**9**). First, intermediate MA–diamine derivatives (**II**, **III**, and **IV**) were prepared, as previously described,^17^ by coupling to the C-28 carboxylic acid group: propane-1,3-diamine, hexane-1,6-diamine, or decane-1,10-diamine (Scheme 1). For the improved efficiency of the amidation reaction between the primary diamine reagents and the C-28 carboxylic acid group of MA, this functionality was activated previously with *O*-(benzotriazol-1-yl)-*N*,*N*,*N*′,*N*′-tetramethyluronium tetrafluoroborate (TBTU). The intermediate MA–TBTU derivative was formed by adding TBTU to a solution of this triterpene in dry THF, in the presence of *N*,*N*-diisopropylethylamine (DIEA), at room temperature for 12 h.^19^ Then, the mixture was diluted with water and extracted with CH_2_Cl_2_, and this organic solution was split into three new solutions. The corresponding diamine reagent (propane-1,3-diamine, hexane-1,6-diamine, or decane-1,10-diamine) was then added in the presence of K_2_CO_3_ to each of the three new solutions, yielding the intermediate MA–diamine derivatives **II**, **III**, and **IV** (Scheme 1).

Three commercial coumarins were used to synthesize nine MA–diamine–coumarin conjugates (**1**–**9**): coumarin-3-carboxylic acid (C3CA), 7-hydroxycoumarinyl-4-acetic acid (HCAA), and *p*-coumaric acid (PCA). Each of these coumarins was dissolved in dioxane, and *N*-(3-(dimethylamino)propyl)-*N*′-ethylcarbodiimide (EDC) was added as a coupling reagent. The three solutions were placed in three reaction tubes of a carousel reaction station and maintained at room temperature for 30 min. Next, each solution was divided into three new solutions (nine tubes and nine solutions). Finally, the corresponding MA–diamine derivative (**II**, **III**, or **IV**) was added, in the presence of K_2_CO_3_, to each of the three different coumarin solutions. These nine solutions were stirred for 2 h at reflux, yielding, respectively, compounds **1**–**9** in very good yields (83–88%; Scheme 1).

Compounds **1**, **2**, and **3** are conjugates of MA with coumarin-3-carboxylic acid (C3CA), through different primary diamine linkers, and gave, respectively, the following molecular masses: 701.4533, 743.5015, and 799.5612 Da. These were compatible with the molecular formulas: C_43_H_60_N_2_O_6_, C_46_H_66_N_2_O_6_, and C_50_H_74_N_2_O_6_, respectively. The ^1^H NMR spectra of these compounds (**1**–**3**) were almost identical, showing the characteristic signals of the triterpene moiety, the proton of the double bond (H-12), and the protons geminal to the hydroxy groups (H-2 and H-3). The distinctive signals of the corresponding diamine chain formed a triplet (ca. δ_H_ 3.00), due to methylene C-1′, linked to the amide group of MA (NH signal between δ_H_ 6.50 and 6.00), and another triplet (ca. δ_H_ 3.45), corresponding to methylene C-3′ (**1**), C-6′ (**2**), or C-10′ (**3**), attached to the amide group of coumarin (NH signal at around δ_H_ 8.85). Finally, the signals of the five aromatic protons of the coumarin skeleton also appeared. The ^13^C NMR spectra of these MA–diamine–coumarin conjugates showed the typical signals of the 30 carbon atoms of the triterpene skeleton and 10 carbon signals of the coumarin moiety, highlighting those of the two amide groups, which were at δ_C_ 178 for the amide group of MA and at 161–162 ppm for the amide group of coumarin. All these data confirmed the structures of the expected MA–diamine–coumarin derivatives (**1**–**3**).

The MA–diamine–coumarin conjugates **4**–**6** were formed by the reaction of the MA–diamine intermediates (**II**, **III**, or **IV**) with hydroxycoumarinyl-4-acetic acid (HCAA). The molecular masses and formulas for these conjugates (**4**–**6**) were, respectively, 731.4640, 773.5108, and 829.5714 Da and C_44_H_62_N_2_O_7_, C_47_H_68_N_2_O_7_, and C_51_H_78_N_2_O_7_. These conjugates were poorly soluble in CDCl_3_, so their NMR spectra were measured in CD_3_OD. The ^1^H NMR spectra of derivatives **4**–**6** were similar, respectively, to those of derivatives **1**–**3**. The ^13^C NMR spectra of these MA–diamine–coumarin conjugates (**4**–**6**) revealed the main differences with respect to those of derivatives **1**–**3** to be the presence of a new methylene carbon signal (C-9″) of the coumarin moiety (11 carbon atoms in total) and a more deshielded quaternary carbon signal (C-7″).

Similarly, the MA–diamine–coumarin conjugates **7**–**9** were formed through the reaction of the MA–diamine intermediates (**II**, **III**, or **IV**) with *p*-coumaric acid (PCA). These conjugate derivatives (**7**–**9**) showed molecular masses and formulas of 675.4734, 717.5211, and 773.5844 Da and C_42_H_62_N_2_O_5_, C_45_H_68_N_2_O_5_, and C_49_H_76_N_2_O_5_, respectively. The ^1^H NMR spectra of **7**–**9** gave characteristic signals of a triterpene moiety, the four aromatic protons of *p*-coumaric acid, and the corresponding diamine chain, highlighting the signals of the two double-bond protons of the coumarin moiety (C-7″ and C-8″) that appeared at 6.4 and 7.5 ppm, respectively. In the ^13^C NMR spectra of **7**–**9**, the signals of the double-bond carbons of the coumarin moiety (C-7″ and C-8″) were notable at δ_C_ 118 and 142, respectively.

Finally, three MA–coumarin conjugates (**10**–**12**) were prepared by coupling either coumarin-3-carboxylic acid (C3CA) to the C-2 and C-3 hydroxy groups of MA, through an ester bond, and/or 7-amino-4-methylcoumarin (AMC) to the C-28 carboxylic acid group of this triterpene compound, through an amide bond (Scheme 2). Thus, when C3CA was added to MA in DMF, in the presence of *N*,*N*′-diisopropylcarbodiimide (DIPCDI) and 4-dimethylaminopyridine (DMAP), the MA–coumarin conjugate **10** was formed. Derivative **10** had two C3CA molecules attached to the C-2 and C-3 hydroxy groups of MA and a molecular mass of 817.3943 Da (C_50_H_56_O_10_). Compound **10** showed typical ^1^H NMR signals of a triterpene and a coumarin moiety, highlighting the deshielded signals of the geminal protons of the ester groups at C-2 and C-3, at δ_H_ 5.47 and 5.20, respectively. MA–coumarin conjugate **11** was formed by treating MA with AMC in dioxane in the presence of EDC. This derivative **11** had an AMC molecule attached to the C-28 carboxyl group of MA, through an amide bond.^23^

MA–coumarin conjugate **12** was synthesized following two different routes, starting from derivative **10** or derivative **11** (Scheme 2). This derivative **12** had two C3CA molecules attached to the C-2 and C-3 hydroxy groups and an AMC molecule linked to the C-28 carboxylic acid group of MA. MA–coumarin conjugate **12** showed a molecular mass of 974.4480 Da (C_60_H_64_NO_11_). The NMR spectra of conjugate **12** were similar to those of derivatives **10** and **11**.

### Effects of MA–Coumarin Conjugates on Cancer Cell Proliferation

Studies were made on the effects of the nine MA–diamine–coumarin conjugates (**1**–**9**) and of the three MA–coumarin derivatives (**10**–**12**) on the proliferation of three cancer cell lines (B16–F10, murine melanoma, HT29, human Caucasian colon adenocarcinoma, and Hep G2, human Caucasian hepatocyte carcinoma). These studies were performed using a 3-(4,5-dimethylthiazol-2-yl)-2,5-diphenyltetrazolium bromide (MTT) assay, with increasing concentration levels of each compound (0–200 μg/mL). Cell viability was determined after treatment for 72 h, by absorption of a formazan dye. These data were expressed as a percentage of untreated control cells. In these three cancer cell lines, the concentration of the compounds that reduced the response by half (IC_50_) was determined (Table 1).

**Table 1:** Growth Inhibitory Effects of MA Conjugates **7** and **10** on B16–F10, HT29, and Hep G2 Cells

compound	B16–F10 (μM)	HT29 (μM)	Hep G2 (μM)
**7**	10.4 ± 0.2	10.9 ± 0.5	8.8 ± 0.4
**10**	0.6 ± 0.0	1.1 ± 0.1	0.9 ± 0.0

a The IC_50_ values (μM) were calculated considering control untreated cells as 100% of viability. Cell-growth inhibition was analyzed by the MTT assay, as described in the Experimental Section.

b Compounds MA (**I**), C3CA, HCAA, PCA, AMC, **1**–**6**, **8**, **9**, **11**, and **12** were inactive (IC_50_ > 10 μM) for all these cell lines.

The four commercial coumarins had very low cytotoxicity, with IC_50_ values higher than 10 μM. The MA–diamine–coumarin conjugates (**1**–**9**), resulting from the link of the MA–diamine intermediates (**II**, **III**, and **IV**) and three of the commercial coumarins (C3CA, HCAA, and PCA), showed a wide range of cytotoxicity, the best results being those for conjugates with the shorter-chain diamine (propane-1,3-diamine), although with IC_50_ values above 10 μM in most cases. The MA-coumarin conjugate (**10**), resulting from the direct link of MA, through the C-2 and C-3 hydroxy groups, with two molecules of coumarin-3-carboxylic acid (C3CA), achieved the best cytotoxicity results, with submicromolar IC_50_ values (0.6, 1.1, and 0.9 μM) in two of the three cell lines assayed. On the other hand, the MA–coumarin conjugates (**11** and **12**), with a 7-amino-4-methylcoumarin (AMC) coupled with the C-28 carboxylic acid group, showed low cytotoxicity, with IC_50_ concentrations also above 10 μM (Table 1).

Only MA conjugates **7** and **10** were selected to compare the growth inhibitory effects in the three nontumor cell lines (HPF, IEC-18, and WRL68) versus the three cancer cell lines (B16–F10, HT29, and Hep G2). Therefore, the cytotoxic effects on B16–F10 murine melanoma cells were compared with those of HPF normal human epithelial lung fibroblasts, with similar tissue. Similarly, the cytotoxic effects of these conjugates on HT29 colon cancer cells and Hep G2 hepatoma cells were compared with those of nontumor cells of the same tissue, such as IEC-18 normal rat ileum cells and WRL68 normal human embryo liver cells, respectively. The percentages of viability of the nontumor cells were calculated using the corresponding IC_50_ values of the MA conjugates (**7** and **10**) for the cancer cells (Table 2). Compound **7** showed cell viability percentages of between 61 and 88% for the nontumor cells, at the corresponding IC_50_ concentration of the respective cancer cell line, while in compound **10**, these cell viability percentages rise to around 90% (Table 2).

**Table 2:** Percentage of Viability of Nontumor Cells at the Corresponding IC_50_ Concentrations for Cancer Cells of MA Conjugates **7** and **10**

compound	percent viability (% viability) of HPF cells	% viability of IEC-18 cells	% viability of WRL68 cells
**7**	76.9 ± 2.9	88.0 ± 0.6	61.4 ± 1.3
**10**	92.9 ± 2.9	89.3 ± 2.2	87.4 ± 1.7

### Characterization of Apoptotic Effects by Flow Cytometry

**Figure 1:**
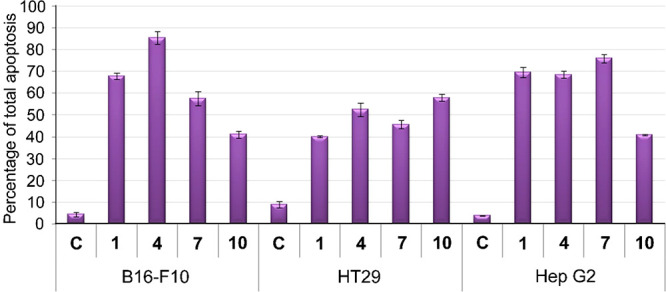
Percentage of total apoptosis of the three cancer cell lines, after exposure to the control (**C**) and the MA conjugates **1**, **4**, **7**, and **10**.

In consideration of the cytotoxic results of these triterpene conjugates with the three cell lines assayed, three MA–diamine–coumarin conjugates (**1**, **4**, and **7**) and the MA–coumarin conjugate **10** were selected for the following cytometry studies on the three cancer cell lines. Thus, the apoptotic determination assays were performed through double staining with Annexin V (An-V), conjugated fluorescein isothiocyanate (FITC), and propidium iodide (PI). The percentages of apoptosis were determined at 72 h, after treatment with the four selected triterpene conjugates (**1**, **4**, **7**, and **10**), at their corresponding IC_50_ concentrations, with Annexin V-FICT/PI by flow-activated cell-sorter (FACS) cytometry analysis. The values of percentage of total apoptosis were expressed as means ± SEM of at least two duplicate experiments (Figure 1).

The four MA conjugates (**1**, **4**, **7**, and **10**) showed apoptotic effects on treated cells, with total apoptosis rates ranging from 40 to 85% relative to the control (Figure 1). The best total apoptosis value on each of the three cancer cell lines was registered by different compounds: **4** for B16–F10 (85%), **10** for HT29 (58%), and **7** for Hep G2 (76%). Furthermore, in all cases, values of the cell necrosis proved low, not exceeding 5–6%. Compound **10**, with the highest inhibition of cell proliferation, was not the most apoptotic, between 41 and 58%. These results appear to indicate that the high cytotoxicity of this derivative occurs through pathways other than apoptosis or that, as this cytotoxicity is so high, there were many dead cells, and information was lost in the flow-cytometry analysis.

### Cell-Cycle Arrest and Distribution

Based on the cytotoxicity shown by derivatives **1**, **4**, **7**, and **10**, a study was made concerning their effects on the distribution of the cell cycle in order to determine possible cytostatic effects caused by the cytotoxic response. The percentages of cells in the different phases of the cycle were analyzed at 72 h, by the incorporation of propidium iodide. Thus, the three cancer cell lines (B16–F10, HT29, and Hep G2) were treated with each of the four MA–coumarin conjugates (**1**, **4**, **7**, and **10**) at their respective IC_50_ concentrations. The values of cell percentage were expressed as means ± SEM of at least two independent experiments performed in triplicate. Flow cytometry was also used to measure DNA ploidy and alterations in cell-cycle profiles. The DNA content is proportional to the fluorescence of propidium iodide, enabling the percentage of cells to be determined in each phase of the cycle and the cell subpopulations with different DNA contents to be visualized.

**Figure 2:**
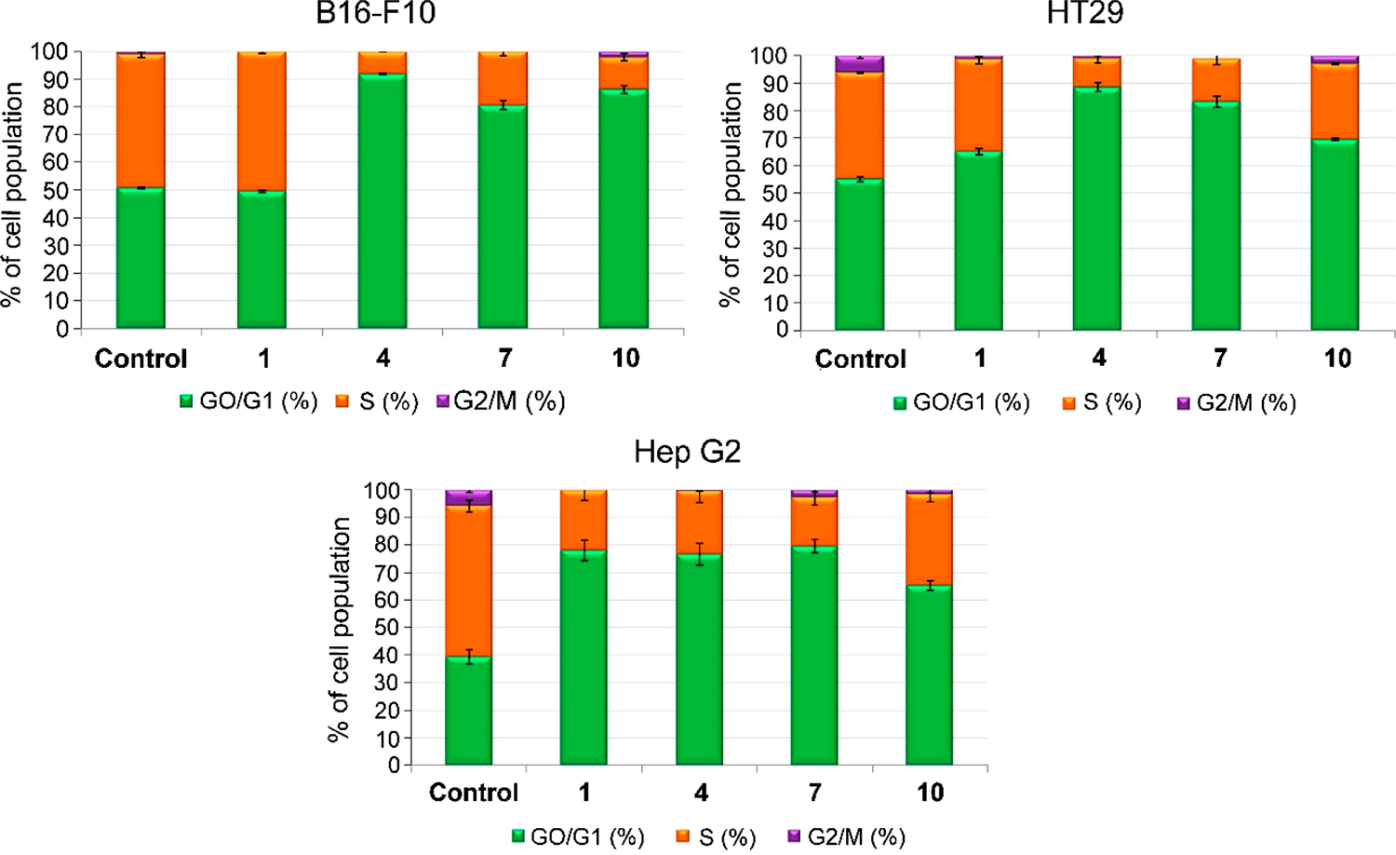
Changes in the cell percentage, in each phase of the cell cycle, of the three cancer cell lines, after exposure to the control and derivatives **1**, **4**, **7**, and **10**.

DNA histogram analysis showed that MA–coumarin conjugates **1**, **4**, **7**, and **10** produced cell-cycle arrest in all three cancer cell lines, increasing the number of cells in the G0/G1 phase, with percentages greater than 70%, except for conjugate **1** in the B16–F10 and HT29 cell lines (Figure 2). These increases in cell population in the G0/G1 phase were accompanied by significant decreases in the percentage of proliferating cells in the S phase, with percentages between 10 and 30%. In all cases, the cell populations in the G2/M phase were practically insignificant (Figure 2).

### Effects on Changes in Mitochondrial-Membrane Potential

**Figure 3:**
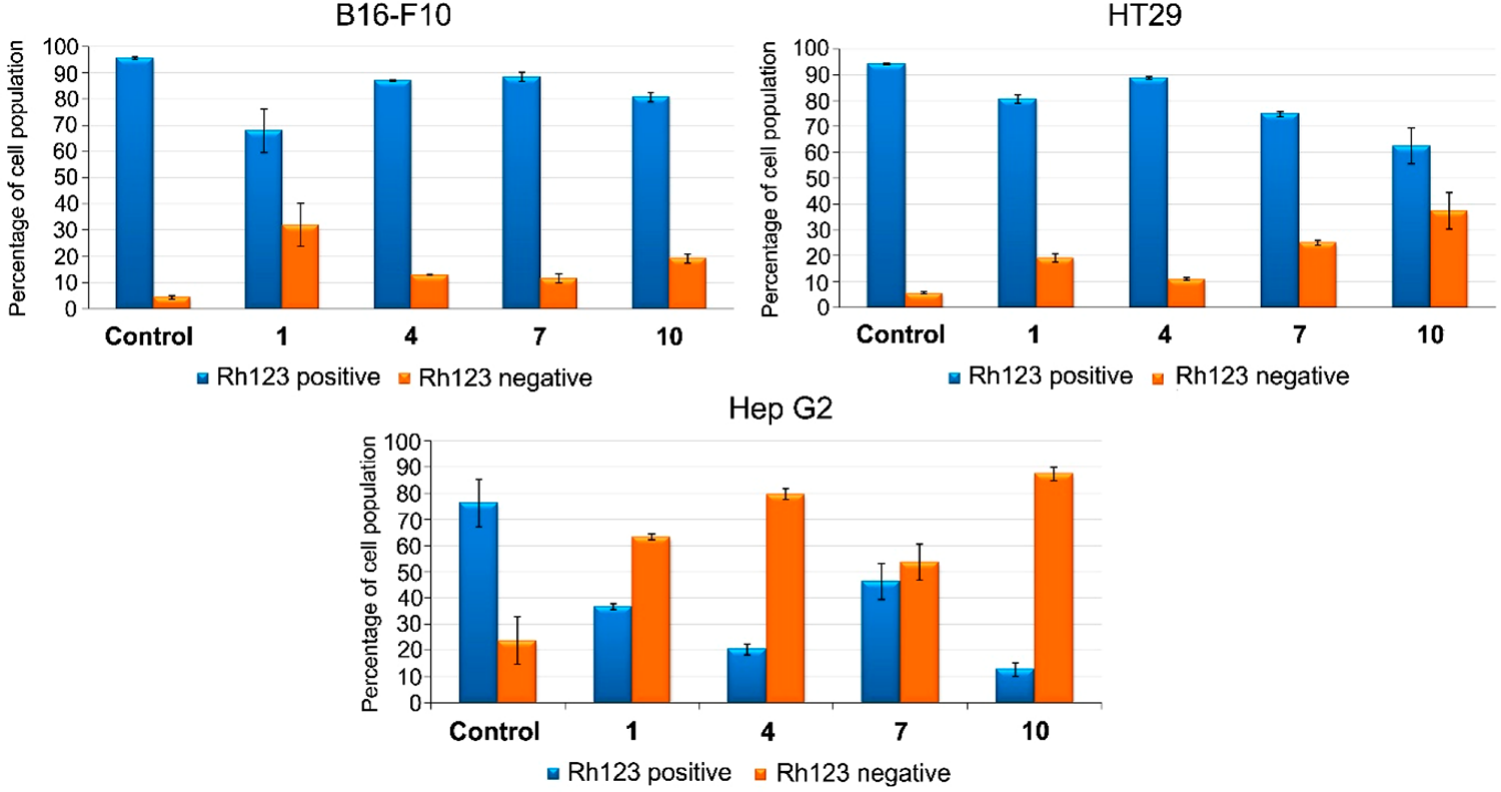
Percentage of cell population of the three cancer cell lines after exposure to the control and derivatives **1**, **4**, **7**, and **10**.

The study of the loss of the mitochondrial-membrane potential (MMP) of tumor cells, when treated with drugs, can provide information on the activation mechanism of apoptosis. This activation can occur extrinsically when MMP is not altered, and intrinsically when a loss of this potential is caused by mitochondrial disruption. The changes in MMP were evaluated in the derivatives **1**, **4**, **7**, and **10** that induced apoptosis in the three cancer cell lines (B16–F10, HT29, Hep G2), at their respective IC_50_ concentrations for 72 h. The values of percentage of cell population were expressed as means ± SEM of at least two experiments in duplicate. Changes in MMP were analyzed by monitoring cell fluorescence after staining with rhodamine 123 (Rh123). The results showed positive Rh123 staining in the B16–F10 and HT29 cell lines for all the compounds tested, suggesting that they activated apoptosis by an extrinsic pathway. However, in the Hep G2 cell line, the compounds showed negative Rh123 staining, disrupting the mitochondrial membrane with a loss of MMP, suggesting the activation of an intrinsic apoptotic pathway, although the mechanism that activated compound **7** was not identified; therefore, further molecular studies are needed (Figure 3).

### Conclusions

The cytotoxicity of several MA–diamine–coumarin conjugates (**1**–**9**) and three MA–coumarin derivatives (**10**–**12**) were evaluated in three cancer cell lines (B16–F10, HT29, and Hep G2), and two of these were deemed as **7** and **10**. The best cytotoxic results were achieved by a derivative of MA (**10**), with two molecules of coumarin-3-carboxylic acid (C3CA) coupled through the C-2 and C-3 hydroxy groups of the triterpene unit. This MA–coumarin conjugate (**10**), with submicromolar IC_50_ values (0.6, 1.1, and 0.9 μM, respectively) in two of the three cancer cell lines, was considerably more effective than its corresponding precursor (MA). Compound **7** showed cell viability percentages of between 61 and 88% for the nontumor cells (HPF, IEC-18, and WRL68), at the corresponding IC_50_ concentration of the respective cancer cell line. The most active MA conjugate (**10**) showed cell viability percentages of around 90% in all nontumor cells.

The four MA conjugates (**1**, **4**, **7**, and **10**) showed apoptotic effects on treated cells, with total apoptosis rates of between 40 and 85%, with necrosis rates that did not exceed 5–6%. The apoptosis results of compound **10** (between 41 and 58%) may be because its high cytotoxicity occurs through pathways other than apoptosis or because there were many dead cells, leading to a loss of information in the flow-cytometry analysis. Almost all the compounds assayed caused cell-cycle arrest in all the cancer cell lines, increasing the number of these cells in the G0/G1 phase (>70%), except for compound **1** in B16–F10 and HT29 cell lines. These increases were accompanied by a decrease in the percentage of proliferating cells in the S phase (10–30%), with the changes in the G2/M phase being less significant.

The cytotoxicity at submicromolar IC_50_ concentrations shown by the MA conjugate by the coumarin-3-carboxylic acid molecules (**10**), in all three cancer cell lines, together with the percentages of cell viability of around 90% in all three nontumor cell lines, suggests its possible future use as a lead compound. This MA–coumarin conjugate could also be utilized as a fluorescent probe that will enable subcellular studies on its location in cells or tissues.

## Experimental Section

### General Experimental Procedures

Melting point (mp) values were determined using a Kofler (Reichter) apparatus and were uncorrected. Optical rotations were measured with a PerkinElmer 241 polarimeter at 25 °C. IR spectra were recorded on a Mattson Satellite FTIR spectrometer. NMR spectra were measured in Varian Inova Unity (300 MHz ^1^H NMR) and Varian Direct Drive (400 and 500 MHz ^1^H NMR) spectrometers. The ^13^C NMR chemical shifts were assigned with the aid of distortionless enhancement by polarization transfer (DEPT) using a flip angle of 135°. The purity of new compounds was determined by a Waters Acquity UPLC system (ultraperformance liquid chromatography), coupled with a Waters Synapt G2 HRMS spectrometer (high-resolution mass spectra) with ESI (electrospray ionization). The purity of all the compounds was confirmed to be ≥95%. Merck silica-gel 60 aluminum sheets (ref 1.16835) were used for TLC, and spots were rendered visible by spraying with H_2_SO_4_–CH_3_COOH, followed by heating to 120 °C and visualization under UV at 254 nm. Merck silica gel 60 (0.040–0.063 mm, ref 1.09385) was used for flash chromatography. CH_2_Cl_2_ (Fisher, ref D/1852/17) with increasing amounts of acetone (Fisher, ref A/0600/17), or *n*-hexane (Merck, ref 1.04374) with increasing amounts of EtOAc (Fisher, ref E/0900/17) or MeOH (Fisher, ref M/4058/17), were used as eluents (all the solvents with analytical reagent-grade purity). The commercial coumarins used were 7-amino-4-methylcoumarin (AMC, CAS Number: 26093-31-2), coumarin-3-carboxylic acid (C3CA, CAS Number: 531-81-7), 7-hydroxycoumarinyl-4-acetic acid (HCAA, CAS Number: 6950-82-9), and *p*-coumaric acid (PCA, CAS Number: 501-98-4) and were purchased from Sigma-Aldrich.

### Isolation of Maslinic Acid (**I**) from Olive Oil Wastes

MA (**I**) was isolated from solid olive oil production wastes, which were extracted successively in a Soxhlet and purified by column chromatography over silica gel, and eluted with CH_2_Cl_2_–acetone or *n*-hexane–EtOAc mixtures of increasing polarity.^25^ These natural compounds can also be extracted more efficiently using an alternative method such as microwave-assisted extraction.^26^

### Synthesis of MA–Diamine Intermediates II, III, and IV

These MA–diamine intermediates were prepared as recommended elsewhere.^17^ Thus, DIEA (0.3 mmol) and TBTU (0.66 mmol) were added to a solution of MA (0.44 mmol) in THF (20 mL). The reaction mixture was maintained at room temperature for 12 h and then diluted with water and extracted three times with CH_2_Cl_2_. The organic layer was dried with anhydrous Na_2_SO_4_, and the solvent was removed under reduced pressure. Finally, the residue was purified by column chromatography using hexane–EtOAc as eluent, yielding the MA–TBTU derivative.^19^ This derivative (0.35 mmol) was dissolved in CH_2_Cl_2_ (15 mL) and divided into three new solutions, whereupon K_2_CO_3_ (1 mmol) and the corresponding diamine reagent (3 mmol of propane-1,3-diamine or hexane-1,6-diamine or decane-1,10-diamine) were added. These reaction mixtures were kept at room temperature for 5 h. Thereafter, CH_2_Cl_2_ was added to each reaction mixture; then, the mixtures were washed several times with water. Each organic layer was treated with anhydrous Na_2_SO_4_, and the solvent was removed under reduced pressure. Finally, each residue was purified in a chromatography column using CH_2_Cl_2_–acetone as the eluent, yielding **II** (90%), **III** (92%), and **IV** (92%), respectively.

### Synthesis of MA–Diamine–Coumarin Conjugates **1**–**9**

Three commercial coumarins, coumarin-3-carboxylic acid (C3CA), 7-hydroxycoumarinyl-4-acetic acid (HCAA), and *p*-coumaric acid (PCA), were placed in three tubes (0.75 mmol each) of a carousel reaction station and dissolved in dioxane (8 mL each), after which EDC (0.75 mmol each) was added. Each reaction mixture was kept at room temperature for 30 min and then divided into three new solutions (9 tubes and 9 solutions). Next, the corresponding MA–diamine intermediate **II**, **III**, or **IV** (0.5 mmol each), in the presence of K_2_CO_3_ (1 mmol each), was added to the three solutions of each of the three coumarins. These nine reaction mixtures were stirred for 2 h at reflux. Then, the mixtures were diluted with water and extracted with CH_2_Cl_2_. The organic layers were treated with anhydrous Na_2_SO_4_. The solvents were removed under reduced pressure, and each residue was purified by column chromatography using CH_2_Cl_2_–acetone (10:1). Derivatives **1**–**9** were obtained in very good yields (83–88%).

#### 2α,3β-Dihydroxy-*N*-(3′-(2″-oxo-2″*H*-chromene-3″-carboxamido)propyl)olean-12-en-28-amide (**1**, 83%)

This compound is a white solid; mp 69–71 °C; [α]^25^_D_ +38 (*c* 1.0, MeOH); IR (film) ν_max_ 2940, 1609, 1531, 1051, 1033 cm^–1^; ^1^H NMR (CDCl_3_, 500 MHz): δ 8.89 (1H, t, *J* = 5.9 Hz, NHCO–coumarin), 8.88 (1H, s, H-4″), 7.69–7.65 (2H, m, H-5″ and H-7″), 7.40–7.36 (2H, m, H-6″ and H-8″), 6.55 (1H, t, *J* = 6.0 Hz, MA-CONH), 5.44 (1H, t, *J* = 3.6 Hz, H-12), 3.65 (1H, ddd, *J* = 9.4, 9.4, 4.3 Hz, H-2), 3.51 and 3.47 (each 1H, m, 2H-3′), 3.39 and 3.09 (each 1H, m, 2H-1′), 2.98 (1H, d, *J* = 9.4 Hz, H-3), 2.67 (1H, dd, *J* = 13.2, 4.2 Hz, H-18), 1.14, 1.00, 0.93, 0.92, 0.89, 0.78, and 0.73 (each 3H, s, Me groups); ^13^C NMR (CDCl_3_, 125 MHz), see Table 3; HRESIMS *m*/*z* 701.4533 (calcd for C_43_H_61_N_2_O_6_ [M + H]^+^, 701.4530).

**Table 3:** ^13^C NMR Spectroscopic Data for Compounds **1**–**9**

	**1**	**2**	**3**	**4**	**5**	**6**	**7**	**8**	**9**
position	δ_C_, type	δ_C_, type	δ_C_, type	δ_C_, type	δ_C_, type	δ_C_, type	δ_C_, type	δ_C_, type	δ_C_, type
1	46.5, CH_2_	46.4, CH_2_	46.7, CH_2_	47.6, CH_2_	47.7, CH_2_	47.7, CH_2_	47.6, CH_2_	47.7, CH_2_	47.7, CH_2_
2	68.9, CH	69.0, CH	68.9, CH	69.5, CH	69.5, CH	69.5, CH	69.5, CH	69.5, CH	69.5, CH
3	83.9, CH	84.0, CH	83.9, CH	84.4, CH	84.4, CH	84.4, CH	84.4, CH	84.4, CH	84.4, CH
4	39.3, C	39.3, C	39.3, C	40.5, C	40.5, C	40.5, C	40.5, C	40.5, C	40.5, C
5	55.3, CH	55.3, CH	55.3, CH	56.6, CH	56.6, CH	56.6, CH	56.6, CH	56.6, CH	56.6, CH
6	18.4, CH_2_	18.4, CH_2_	18.5, CH_2_	19.5, CH_2_	19.5, CH_2_	19.6, CH_2_	19.6, CH_2_	19.5, CH_2_	19.6, CH_2_
7	32.4, CH_2_	32.4, CH_2_	32.5, CH_2_	33.7, CH_2_	33.8, CH_2_	33.8, CH_2_	33.7, CH_2_	33.8, CH_2_	33.8, CH_2_
8	39.5, C	39.5, C	39.6, C	40.7, C	40.7, C	40.7, C	40.7, C	40.7, C	40.7, C
9	47.6, CH	47.6, CH	47.7, CH	48.9, CH	49.0, CH	48.9, CH	49.0, CH	49.0, CH	49.0, CH
10	38.3, C	38.3, C	38.4, C	39.2, C	39.2, C	39.2, C	39.2, C	39.2, C	39.2, C
11	23.7, CH_2_	23.7, CH_2_	23.8, CH_2_	23.9, CH_2_	24.0, CH_2_	24.0, CH_2_	24.0, CH_2_	24.0, CH_2_	24.0, CH_2_
12	125.4, CH	125.4, CH	125.4, CH	123.9, CH	123.8, CH	123.7, CH	123.9, CH	123.8, CH	123.7, CH
13	144.7, C	145.2, C	145.5, C	145.2, C	145.5, C	145.5, C	145.3, C	145.5, C	145.5, C
14	42.1, C	42.2, C	42.3, C	42.9, C	43.0, C	43.0, C	42.9, C	43.0, C	43.0, C
15	29.7, CH_2_	29.5, CH_2_	29.8, CH_2_	30.2, CH_2_	30.4, CH_2_	30.6, CH_2_	30.3, CH_2_	30.5, CH_2_	30.7, CH_2_
16	23.7, CH_2_	23.7, CH_2_	23.8, CH_2_	24.6, CH_2_	24.6, CH_2_	24.7, CH_2_	24.7, CH_2_	24.7, CH_2_	24.7, CH_2_
17	46.4, C	46.6, C	46.4, C	47.6, C	47.5, C	47.5, C	47.6, C	47.5, C	47.5, C
18	41.9, CH	42.4, CH	42.6, CH	42.5, CH	42.6, CH	42.6, CH	42.5, CH	42.6, CH	42.7, CH
19	46.8, CH_2_	46.9, CH_2_	46.9, CH_2_	48.1, CH_2_	48.1, CH_2_	48.1, CH_2_	48.1, CH_2_	48.1, CH_2_	48.1, CH_2_
20	30.9, C	30.9, C	30.9, C	31.6, C	31.6, C	31.6, C	31.6, C	31.6, C	31.6, C
21	34.3, CH_2_	34.2, CH_2_	34.3, CH_2_	35.1, CH_2_	35.1, CH_2_	35.1, CH_2_	35.1, CH_2_	35.1, CH_2_	35.1, CH_2_
22	33.0, CH_2_	32.6, CH_2_	32.7, CH_2_	34.4, CH_2_	34.4, CH_2_	34.3, CH_2_	34.4, CH_2_	34.4, CH_2_	34.3, CH_2_
23	28.7, CH_3_	28.7, CH_3_	28.8, CH_3_	29.3, CH_3_	29.3, CH_3_	29.3, CH_3_	29.3, CH_3_	29.3, CH_3_	29.3, CH_3_
24	16.8, CH_3_	16.9, CH_3_	16.9, CH_3_	17.5, CH_3_	17.5, CH_3_	17.5, CH_3_	17.4, CH_3_	17.5, CH_3_	17.5, CH_3_
25	16.8, CH_3_	16.9, CH_3_	16.9, CH_3_	17.1, CH_3_	17.1, CH_3_	17.2, CH_3_	17.1, CH_3_	17.1, CH_3_	17.2, CH_3_
26	17.1, CH_3_	17.1, CH_3_	17.2, CH_3_	17.8, CH_3_	18.0, CH_3_	18.1, CH_3_	17.9, CH_3_	18.0, CH_3_	18.1, CH_3_
27	25.9, CH_3_	25.9, CH_3_	25.9, CH_3_	26.5, CH_3_	26.5, CH_3_	26.5, CH_3_	26.5, CH_3_	26.4, CH_3_	26.5, CH_3_
28	178.3, C	178.4, C	178.1, C	180.4, C	180.2, C	180.1, C	180.5, C	180.3, C	180.2, C
29	23.7, CH_3_	23.7, CH_3_	25.9, CH_3_	24.0, CH_3_	24.0, CH_3_	24.0, CH_3_	24.1, CH_3_	24.0, CH_3_	24.0, CH_3_
30	33.2, CH_3_	33.1, CH_3_	33.2, CH_3_	33.6, CH_3_	33.6, CH_3_	33.6, CH_3_	33.6, CH_3_	33.5, CH_3_	33.6, CH_3_
1′	37.0, CH_2_	39.9, CH_2_	40.2, CH_2_	37.5, CH_2_	40.5, CH_2_	40.7, CH_2_	37.8, CH_2_	40.5, CH_2_	40.8, CH_2_
2′	27.5, CH_2_	29.3, CH_2_	29.6, CH_2_	28.5, CH_2_	28.5, CH_2_	30.5, CH_2_	28.5, CH_2_	28.5, CH_2_	30.5, CH_2_
3′	36.2, CH_2_	26.9, CH_2_	29.6, CH_2_	37.5, CH_2_	27.6, CH_2_	28.5, CH_2_	37.8, CH_2_	27.8, CH_2_	28.4, CH_2_
4′		27.4, CH_2_	29.6, CH_2_		27.6, CH_2_	30.5, CH_2_		27.8, CH_2_	30.5, CH_2_
5′		26.9, CH_2_	29.6, CH_2_		30.3, CH_2_	30.5, CH_2_		30.4, CH_2_	30.5, CH_2_
6′		39.6, CH_2_	29.6, CH_2_		40.5, CH_2_	30.5, CH_2_		40.5, CH_2_	30.5, CH_2_
7′			27.4, CH_2_			28.1, CH_2_			28.4, CH_2_
8′			27.4, CH_2_			28.1, CH_2_			28.1, CH_2_
9′			27.4, CH_2_			30.5, CH_2_			30.5, CH_2_
10′			39.6, CH_2_			40.7, CH_2_			40.8, CH_2_
1″							127.7, C	127.8, C	127.7, C
2″	161.5, C	161.6, C	161.6, C	163.4, C	163.4, C	163.4, C	130.6, CH	130.5, CH	130.5, CH
3″	118.4, C	118.6, C	118.7, C	113.2, CH	113.0, CH	113.0, CH	116.8, CH	116.7, CH	116.7, CH
4″	148.5, CH	148.4, CH	148.4, CH	152.5, C	152.7, C	152.7, C	160.6, C	160.5, C	160.5, C
4a″	118.7, C	118.8, C	118.8, C	113.1, C	113.1, C	113.1, C			
5″	129.9, CH	129.9, CH	130.0, CH	127.5, CH	127.5, CH	127.5, CH	116.8, CH	116.7, CH	116.7, CH
6″	122.7, CH	122.7, CH	122.6, CH	114.4, CH	114.3, CH	114.4, CH	130.6, CH	130.5, CH	130.5, CH
7″	134.3, CH	134.2, CH	134.1, CH	163.1, C	163.1, C	163.1, C	142.0, CH	141.7, CH	141.7, CH
8″	116.8, CH	116.7, CH	116.8, CH	103.8, CH	103.7, CH	103.7, CH	118.3, CH	118.5, CH	118.5, CH
8a″	154.5, C	154.5, C	154.6, C	156.8, C	156.8, C	156.8, C			
9″	162.2, C	161.6, C	161.7, C	37.8, CH_2_	40.3, CH_2_	40.3, CH_2_	169.4, C	169.2, C	169.2, C
10″				171.0, C	170.7, C	170.7, C			

#### 2α,3β-Dihydroxy-*N*-(6′-(2″-oxo-2″*H*-chromene-3″-carboxamido)hexyl)olean-12-en-28-amide (**2**, 85%)

This compound is a white solid; mp 73–75 °C; [α]^25^_D_ +12 (*c* 1.0, MeOH); IR (film) ν_max_ 2967, 1614, 1567, 1168, 1033 cm^–1^; ^1^H NMR (CDCl_3_, 400 MHz): δ 8.90 (1H, s, H-4″), 8.83 (1H, t, *J* = 5.9 Hz, NHCO–coumarin), 7.70–7.64 (2H, m, H-5″ and H-7″), 7.41–7.36 (2H, m, H-6″ and H-8″), 5.97 (1H, t, *J* = 6.7 Hz, MA–CONH), 5.38 (1H, t, *J* = 3.6 Hz, H-12), 3.67 (1H, ddd, *J* = 9.4, 9.4, 4.3 Hz, H-2), 3.45 (2H, m, 2H-6′), 3.34 and 2.99 (each 1H, m, 2H-1′), 2.99 (1H, d, *J* = 9.4 Hz, H-3), 2.49 (1H, dd, *J* = 13.2, 4.2 Hz, H-18), 1.14, 1.01, 0.97, 0.89, 0.89, 0.80, and 0.75 (each 3H, s, Me groups); ^13^C NMR (CDCl_3_, 100 MHz), see Table 3; HRESIMS *m*/*z* 743.5015 (calcd for C_46_H_67_N_2_O_6_ [M + H]^+^, 743.4999).

#### 2α,3β-Dihydroxy-*N*-(10′-(2″-oxo-2″*H*-chromene-3″-carboxamido)decyl)olean-12-en-28-amide (**3**, 86%)

This compound is a white solid; mp 86–88 °C; [α]^25^_D_ +18 (*c* 1.0, MeOH); IR (film) ν_max_ 2923, 1708, 1455, 1053, 1033 cm^–1^; ^1^H NMR (CDCl_3_, 400 MHz): δ 8.90 (1H, s, H-4″), 8.83 (1H, t, *J* = 5.8 Hz, NHCO–coumarin), 7.70–7.64 (2H, m, H-5″ and H-7″), 7.41–7.36 (2H, m, H-6″ and H-8″), 5.90 (1H, t, *J* = 5.5 Hz, MA–CONH), 5.38 (1H, t, *J* = 3.6 Hz, H-12), 3.71 (1H, ddd, *J* = 11.3, 9.5, 4.5 Hz, H-2), 3.45 (2H, c, *J* = 6.7, 5.8 Hz, 2H-10′), 3.34 and 3.01 (each 1H, m, 2H-1′), 2.99 (1H, d, *J* = 9.5 Hz, H-3), 2.49 (1H, dd, *J* = 13.2, 4.3 Hz, H-18), 1.16, 1.03, 0.99, 0.90, 0.90, 0.82, and 0.77 (each 3H, s, Me groups); ^13^C NMR (CDCl_3_, 100 MHz), see Table 3; HRESIMS *m*/*z* 799.5612 (calcd for C_50_H_75_N_2_O_6_ [M + H]^+^, 799.5625).

#### 2α,3β-Dihydroxy-*N*-(3′-(2″-(7‴-hydroxy-2‴-oxo-2‴*H*-chromen-4‴-yl)acetamido) propyl)olean-12-en-28-amide (**4**, 87%)

This compound is a white solid; mp 143–145 °C; [α]^25^_D_ +16 (*c* 1.0, MeOH); IR (film) ν_max_ 2938, 1607, 1455, 1052, 1033 cm^–1^; ^1^H NMR (CD_3_OD, 500 MHz): δ 7.63 (1H, d *J* = 8.7 Hz, H-5″), 7.33 (1H, t, *J* = 5.9 Hz, MA–CONH), 6.86 (1H, dd, *J* = 8.7, 2.4 Hz, H-6″), 6.71 (1H, d, *J* = 2.4 Hz, H-8″), 6.21 (1H, s, H-3″), 5.34 (1H, t, *J* = 3.7 Hz, H-12), 3.73 (1H, d, *J* = 8.0 Hz, H-9″), 3.62 (1H, ddd, *J* = 11.3, 9.2, 4.4 Hz, H-2), 3.30–3.00 (4H, m, 2H-1′ and 2H-3′), 2.91 (1H, d, *J* = 9.2 Hz, H-3), 2.81 (1H, dd, *J* = 13.6, 4.5 Hz, H-18), 1.14, 1.01, 0.94, 0.92, 0.90, 0.80, and 0.67 (each 3H, s, Me groups); ^13^C NMR (CD_3_OD, 125 MHz), see Table 3; HRESIMS *m*/*z* 731.4640 (calcd for C_44_H_63_N_2_O_7_ [M + H]^+^, 731.4635).

#### 2α,3β-Dihydroxy-*N*-(6′-(2″-(7‴-hydroxy-2‴-oxo-2‴*H*-chromen-4‴-yl)acetamido)hexyl)olean-12-en-28-amide (**5**, 84%)

This compound is a white solid; mp 139–141 °C; [α]^25^_D_ +5 (*c* 1.0, MeOH); IR (film) ν_max_ 2937, 1607, 1466, 1053, 1033 cm^–1^; ^1^H NMR (CD_3_OD, 500 MHz): δ 7.61 (1H, d, *J* = 8.8 Hz, H-5″), 6.81 (1H, dd, *J* = 8.8, 2.4 Hz, H-6″), 6.73 (1H, d, *J* = 2.4 Hz, H-8″), 6.21 (1H, s, H-3″), 5.35 (1H, t, *J* = 3.7 Hz, H-12), 3.73 (2H, s, H-9″), 3.61 (1H, ddd, *J* = 11.3, 9.2, 4.4 Hz, H-2), 3.25–3.00 (4H, m, 2H-1′ and 2H-6′), 2.91 (1H, d, *J* = 9.2 Hz, H-3), 2.79 (1H, dd, *J* = 13.4, 4.4 Hz, H-18), 1.17, 1.00, 0.98, 0.94, 0.91, 0.79, and 0.77 (each 3H, s, Me groups); ^13^C NMR (CD_3_OD, 125 MHz), see Table 3; HRESIMS *m*/*z* 773.5108 (calcd for C_47_H_69_N_2_O_7_ [M + H]^+^, 773.5105).

#### 2α,3β-Dihydroxy-*N*-(10′-(2″-(7‴-hydroxy-2‴-oxo-2‴*H*-chromen-4‴-yl)acetamido) decyl)olean-12-en-28-amide (**6**, 86%)

This compound is a white solid; mp 120–122 °C; [α]^25^_D_ +27 (*c* 1.0, MeOH); IR (film) ν_max_ 2923, 1709, 1607, 1053, 1033 cm^–1^; ^1^H NMR (CD_3_OD, 400 MHz): δ 7.62 (1H, d, *J* = 8.8 Hz, H-5″), 6.81 (1H, dd, *J* = 8.7, 2.4 Hz, H-6″), 6.72 (1H, d, *J* = 2.4 Hz, H-8″), 6.21 (1H, s, H-3″), 5.36 (1H, t, *J* = 3.5 Hz, H-12), 3.73 (1H, d, *J* = 8.0 Hz, H-9″), 3.62 (1H, ddd, *J* = 11.3, 9.5, 4.5 Hz, H-2), 3.30–3.00 (4H, m, 2H-1′and 2H-10′), 2.92 (1H, d, *J* = 9.5 Hz, H-3), 2.80 (1H, dd, *J* = 13.1, 4.3 Hz, H-18), 1.17, 1.01, 0.99, 0.94, 0.91, 0.80, and 0.78 (each 3H, s, Me groups); ^13^C NMR (CD_3_OD, 100 MHz), see Table 3; HRESIMS *m*/*z* 829.5714 (calcd for C_51_H_77_N_2_O_7_ [M + H]^+^, 829.5731).

#### 2α,3β-Dihydroxy-*N*-(3′-((*E*)-3″-(4‴-hydroxyphenyl)acrylamido)propyl)olean-12-en-28-amide (**7**, 83%)

This compound is a white solid; mp 123–125 °C; [α]^25^_D_ +33 (*c* 1.0, MeOH); IR (film) ν_max_ 2938, 1604, 1514, 1053, 1033 cm^–1^; ^1^H NMR (CD_3_OD, 500 MHz): δ 7.47 (1H, d, *J* = 15.7 Hz, H-7″), 7.41 (2H, d, *J* = 8.6 Hz, H-3″ and H-5″), 6.79 (2H, d, *J* = 8.6 Hz, H-2″and H-6″), 6.42 (1H, d, *J* = 15.7 Hz, H-8″), 5.41 (1H, t, *J* = 3.6 Hz, H-12), 3.59 (1H, ddd, *J* = 11.3, 9.5 4.5 Hz, H-2), 3.37–3.13 (4H, m, 2H-1′ and 2H-3′), 2.89 (1H, d, *J* = 9.5 Hz, H-3), 2.83 (1H, dd, *J* = 13.4, 4.4 Hz, H-18), 1.17, 0.99, 0.96, 0.94, 0.91, 0.75, and 0.75 (each 3H, s, Me groups); ^13^C NMR (CD_3_OD, 125 MHz), see Table 3; HRESIMS *m*/*z* 675.4734 (calcd for C_42_H_63_N_2_O_5_ [M + H]^+^, 675.4737).

#### 2α,3β-Dihydroxy-*N*-(6′-((*E*)-3″-(4‴-hydroxyphenyl)acrylamido)hexyl)olean-12-en-28-amide (**8**, 88%)

This compound is a white solid; mp 76–78 °C; [α]^25^_D_ +17 (*c* 1.0, MeOH); IR (film) ν_max_ 2927, 1602, 1513, 1168, 1033 cm^–1^; ^1^H NMR (CD_3_OD, 400 MHz): δ 7.45 (1H, d, *J* = 15.7 Hz, H-7″), 7.40 (2H, d, *J* = 8.6 Hz, H-3″ and H-5″), 6.79 (2H, d, *J* = 8.6 Hz, H-2″and H-6″), 6.41 (1H, d, *J* = 15.7 Hz, H-8″), 5.35 (1H, t, *J* = 3.6 Hz, H-12), 3.60 (1H, ddd, *J* = 11.3, 9.6 4.5 Hz, H-2), 3.29 (2H, m, 2H-1′), 3.21 and 3.09 (each 1H, m, 2H-6′), 2.89 (1H, d, *J* = 9.6 Hz, H-3), 2.83 (1H, dd, *J* = 13.2, 4.3 Hz, H-18), 1.17, 0.99, 0.98, 0.94, 0.90, 0.78, and 0.78 (each 3H, s, Me groups); ^13^C NMR (CD_3_OD, 100 MHz), see Table 3; HRESIMS *m*/*z* 717.5211 (calcd for C_45_H_69_N_2_O_5_ [M + H]^+^, 717.5206).

#### 2α,3β-Dihydroxy-*N*-(10′-((*E*)-3″-(4‴-hydroxyphenyl)acrylamido)decyl)olean-12-en-28-amide (**9**, 84%)

This compound is a white solid; mp 104–106 °C; [α]^25^_D_ +22 (*c* 1.0, MeOH); IR (film) ν_max_ 2923, 1604, 1513, 1053, 1033 cm^–1^; ^1^H NMR (CD_3_OD, 400 MHz): δ 7.45 (1H, d, *J* = 15.7 Hz, H-7″), 7.41 (2H, d, *J* = 8.6 Hz, H-3″ and H-5″), 6.79 (2H, d, *J* = 8.6 Hz, H-2″and H-6″), 6.41 (1H, d, *J* = 15.7 Hz, H-8″), 5.35 (1H, t, *J* = 3.5 Hz, H-12), 3.62 (1H, ddd, *J* = 11.3, 9.6 4.4 Hz, H-2), 3.28 (2H, t, *J* = 7.2 Hz, 2H-1′), 3.19 and 3.07 (each 1H, m, 2H-10′), 2.90 (1H, d, *J* = 9.6 Hz, H-3), 2.78 (1H, dd, *J* = 13.4, 4.2 Hz, H-18), 1.16, 1.01, 1.00, 0.94, 0.90, 0.80, and 0.78 (each 3H, s, Me groups); ^13^C NMR (CD_3_OD, 100 MHz), see Table 3; HRESIMS *m*/*z* 773.5844 (calcd for C_49_H_77_N_2_O_5_ [M + H]^+^, 773.5832).

### Synthesis of MA–Coumarin Conjugate **10**

MA (**I**) (0.5 mmol) in DMF (5 mL) was treated with coumarin-3-carboxylic acid (C3CA, 3 mmol) in the presence of DIPCDI (3 mmol) and DMAP (3 mmol). The reaction mixture was kept at 60 °C for 12 h and then diluted with water and extracted with CH_2_Cl_2_. Afterward, the organic layer was dried with anhydrous Na_2_SO_4_. The solvent was removed under reduced pressure, and the residue was purified by column chromatography using CH_2_Cl_2_–acetone (10:1) to give compound **10** (80%).

#### 2α,3β-bis((2′-oxo-2′*H*-Chromene-3″-carbonyl)oxy)-olean-12-en-28-amide (**10**, 80%)

This compound is a white solid; mp 200–202 °C; [α]^25^_D_ −64 (*c* 1.0, MeOH); IR (film) ν_max_ 2939, 1609, 1455, 1055, 1033 cm^–1^; ^1^H NMR (CDCl_3_, 500 MHz) and ^13^C NMR (CDCl_3_, 125 MHz), see Table 4; HRESIMS *m*/*z* 817.3943 (calcd for C_50_H_57_O_10_ [M + H]^+^, 817.3952).

**Table 4:** ^13^C NMR Spectroscopic Data for Compounds **10** and **12**

	**10**			**12**
position	δ_C_, type	δ_H_, (*J* in Hz)	HMBC^a^	δ_C_, type
1	43.9, CH_2_	2.28, dd (12.4, 4.6), 1.26, m	2, 3, 5, 9, 10, 25	43.9, CH_2_
2	71.8, CH	5.47, ddd (10.3, 10.3, 4.6)	1, 3, 9′	71.6, CH
3	81.9, CH	5.20, d (10.3)	1, 2, 4, 23, 24, 9″	81.7, CH
4	39.9, C	1.12, m	3, 4, 6, 10, 23, 24, 25	39.8, C
5	55.2, CH	1.64, 1.49 m	4, 5, 7	54.9, CH
6	18.4, CH_2_	1.51, 1.37, m	5, 8, 9, 26	18.2, CH_2_
7	32.6, CH_2_	1.70, m	1, 8, 10, 14, 25, 26	32.2, CH_2_
8	39.5, C	1.98, 1.92, m	8, 9, 12, 13	39.5, C
9	47.7, CH	5.29, t (3.7)	9, 11, 14	47.5, CH
10	38.6, C	1.75, 1.11, m	17, 27	38.3, C
11	22.9, CH_2_	1.98, 1.65, m	15, 17, 28	23.9, CH_2_
12	122.3, CH	2.84, dd (13.7, 4.7)	12, 13, 14, 16, 17, 19, 28	123.2, CH
13	143.8, C	1.61, 1.16, m	13, 17, 18, 20, 29, 30	145.1, C
14	41.7, C	1.34, 1.22, m	20, 22, 29	42.5, C
15	27.8, CH_2_	1.78, 1.57, m	16, 17, 21	27.3, CH_2_
16	23.7, CH_2_	1.07, s	3, 4, 5, 24	24.2, CH_2_
17	46.6, C	1.10, s	3, 4, 5, 23	47.5, C
18	41.0, CH	1.16, s	1, 5, 9, 10	42.3, CH
19	45.9, CH_2_	0.78, s	7, 8, 9, 14	46.7, CH_2_
20	30.8, C	1.15, s	8, 13, 14, 15	30.8, C
21	33.9, CH_2_	0.93, s	19, 20, 21, 30	34.1, CH_2_
22	32.6, CH_2_	0.90, s	19, 20, 21, 29	32.2, CH_2_
23	28.7, CH_3_	8.45, s	2′, 3′, 4a′, 5′, 8a′, 9′	28.6, CH_3_
24	18.0, CH_3_	8.40, s	2″, 3″, 4a″, 5″, 8a″, 9″	17.9, CH_3_
25	16.6, CH_3_	7.71, dd (7.7, 1.6)	4′, 4a′, 7′, 8a′	16.5, CH_3_
26	17.3, CH_3_	7.61, m	4″, 7″, 8a″	16.9, CH_3_
27	26.1, CH_3_	7.32, ddd (7.7, 7.7, 1.1)	4a′, 5′, 7′, 8′	25.8, CH_3_
28	184.2, C	7.30, m	4a″, 5″, 7″, 8″	176.9, C
29	23.7, CH_3_	7.60, m	5′, 8a′	23.6, CH_3_
30	33.2, CH_3_	7.60, m	5″, 8a″	32.9, CH_3_
2′	156.8, C	7.28, m	4a′, 6′, 8a′	156.7, C
2″	156.8, C	7.28, m	4a″, 6″, 8a″	156.6, C
3′	117.9, C			117.8, C
3″	117.5, C			117.4, C
4′	148.9, CH			148.8, CH
4″	148.0, CH			147.9, CH
4a′	118.3, C			118.1, C
4a″	118.0, C			117.9, C
5′	130.3, CH			130.2, CH
5″	129.9, CH			129.7, CH
6′	134.5, CH			134.4, CH
6″	134.5, CH			134.4, CH
7′	125.0, CH			124.9, CH
7″	125.0, CH			124.9, CH
8′	116.8, CH			116.7, CH
8″	116.7, CH			116.6, CH
8a′	155.2, C			155.0, C
8a″	155.1, C			155.0, C
9′	162.7, C			162.5, C
9″	162.0, C			161.8, C
2‴				161.0, C
3‴				113.5, CH
4‴				152.1, C
4a‴				116.1, C
5‴				125.2, CH
6‴				115.6, CH
7‴				141.3, C
8‴				107.2, CH
8a‴				154.3, C
9‴				18.6, CH_3_

a HMBC correlations, optimized for 8 Hz, are from the proton(s) stated to the indicated carbon.

### Synthesis of MA–Coumarin Conjugate **11**

Compound **11** was synthesized as published elsewhere.^23^ MA (0.42 mmol) was dissolved in anhydrous CH_2_Cl_2_ (10 mL); then, 1-ethyl-3-(3-(dimethylamino)propyl)carbodiimide (EDC, 0.63 mmol) and 7-amino-4-methylcoumarin (AMC, 0.42 mmol) were added. After the workup, 2α,3β-dihydroxy-*N*-(4-methyl-2-oxo-2*H*-chromen-7-yl)-olean-12-en-28-amide (**11**, 90%) resulted.^23^

### Synthesis of MA–Coumarin Conjugate **12**

Conjugate **12** was synthesized by two different pathways. We first started with compound **10**. This compound (0.5 mmol) was dissolved in dioxane (10 mL) and treated with amino-4-methylcoumarin (AMC, 0.75 mmol) and EDC (0.75 mmol) for 2 h at reflux. After the normal workup and purification on a chromatography column, compound **12** (85%) resulted. The second pathway started with compound **11**. This compound (0.5 mmol) was treated with coumarin-3-carboxylic acid (C3CA, 3 mmol) in the presence of DIPCDI (3 mmol) and DMAP (3 mmol) at 60 °C for 12 h. The usual workup and purification in a chromatography column gave compound **12** (87%).

#### *N*-(4‴-Methyl-2‴-oxo-2‴*H*-chromen-7‴-yl)-2α,3β-bis((2′-oxo-2′*H*-chromene-3′-carbonyl)oxy)-olean-12-en-28-amide (**12**, 85–87%)

This compound is a white solid; mp 206–208 °C; [α]^25^_D_ −20 (*c* 1.0, MeOH); IR (film) ν_max_ 2967, 1713, 1608, 1055, 1033 cm^–1^; ^1^H NMR (CDCl_3_, 400 MHz): δ 8.45 (1H, s H-4′), 8.38 (1H, s H-4″), 7.88 (1H, br s, NH), 7.70–7.25 (11H, m, H-5′, H-5″, H-6′, H-6″, H-7′, H-7″, H-8′, H-8″, H-5‴, H-6‴, and H-8‴), 6.20 (1H, br s, H-3‴), 5.58 (1H, t, *J* = 3.6 Hz, H-12), 5.46 (1H, ddd, *J* = 10.7, 10.7, 4.5 Hz, H-2), 5.19 (1H, d, *J* = 10.7 Hz, H-3), 2.69 (1H, dd, *J* = 12.1, 2.5, H-18), 2.41 (3H, br s, H-9‴), 1.23, 1.13, 1.07, 1.05, 0.95, 0.95, and 0.71 (each 3H, s, Me groups); ^13^C NMR (CDCl_3_, 100 MHz), see Table 4; HRESIMS *m*/*z* 974.4480 (calcd for C_60_H_64_NO_11_ [M + H]^+^, 974.4479).

### Test Compounds

The MA derivatives employed in biological treatments were dissolved before use at a concentration of 5 mg/mL in DMSO. They were stored at −20 °C, constituting a stock solution from which other solutions were made for the different tests. Prior to the experiments, this solution was diluted in cell-culture medium.

### Cell Cultures

B16–F10 murine melanoma cells (ATTC CRL-6475), HT29 human Caucasian colon adenocarcinoma cells (ECACC 9172201; ATTC HTB-38), Hep G2 human Caucasian hepatocyte carcinoma cells (ECACC 85011430), nontumor human epithelial lung fibroblast cells HPF (ScienCell catalog (cat.) no. 3300), nontumor rat ileum cells IEC-18 (ECACC no. 88011801), and nontumor human embryo liver cells (ECACC 89121403) were cultured in DMEM medium supplemented with 2 mM glutamine, 10% heat-inactivated fetal bovine serum (FCS), 10.000 units/mL penicillin, and 10 mg/mL streptomycin. These cells were incubated at 37 °C in an atmosphere of 5% CO_2_ and 95% humidity. The culture media were changed every 48 h, and the confluent cultures were separated with a trypsin solution (0.25% EDTA). Subconfluent monolayer cells were used in all experiments. All cell lines used were provided by the cell bank of the University of Granada (Spain).

### Cell Proliferation Activity Assay

The effect of the synthesized compounds on the viability of tumor cells was assessed using an MTT assay, based on the ability of living cells to cleave the tetrazolium ring, thus producing formazan, which absorbs at 570 nm. The MA conjugates (**1**–**12**) were assayed against the selected cancer cell lines. For an evaluation of the cytotoxic effects of these compounds, the cells were seeded in 96-well plates at an initial density of 5 × 10^3^ B16–F10 cells, 6 × 10^3^ HT29 cells, 15 × 10^3^ Hep G2 cells, 8 × 10^3^ HPF cells, 15 × 10^3^ IEC-18 cells, and 11 × 10^3^ WRL68 cells, per well. After incubation for 24 h, the cells were treated with the synthesized compounds in triplicate, at different concentrations (0–200 μg/mL), and incubated for 72 h. Thereafter, 100 μL of MTT solution (0.5 mg/mL) were added to each well. After 2 h of incubation, the cells were washed twice with phosphate buffered saline (PBS), and the formazan was resuspended in 100 μL of DMSO. Relative cell viability, with respect to untreated control cells, was measured by absorbance at 550 nm on an ELISA plate reader (Tecan Sunrise MR20-301, TECAN, Austria). Four compounds with low IC_50_ values (**1**, **4**, **7**, and **10**) were selected for several cytometry assays such as apoptosis and cell cycle. These experiments were measured and compared to the control after 72 h of treatment.

### Annexin V-FICT/Propidium Iodide Flow-Cytometry Analysis

Apoptosis was assessed by flow cytometry using a FACScan (fluorescence-activated cell sorter) flow cytometer (Coulter Corporation, Hialeah, FL, USA). For these assays, 5 × 10^4^ B16–F10, 6 × 10^4^ HT29 cells, and 15 × 10^4^ Hep G2 cells were placed in 24-well plates with 1.5 mL of medium and incubated for 24 h. Subsequently, the cells were treated with the selected compounds in triplicate for 72 h at their corresponding IC_50_ concentrations. The cells were collected and resuspended in a binding buffer (10 mM HEPES/NaOH, pH 7.4, 140 mM NaCl, 2.5 mM CaCl_2_). Annexin V-FITC conjugate (1 mg/mL) was then added and incubated for 15 min at room temperature in darkness. Just before the analysis by flow cytometry, cells were stained with 20 μL of 1 mg/mL PI solution. In each experiment, approximately 10 × 10^3^ cells were analyzed, and the experiment was duplicated twice.

### Cell Cycle

The method used to quantify the amount of DNA in the different phases of the cell cycle (G0/G1, S, and G2/M) was performed by flow cytometry, using a fluorescence-activated cell sorter (FACS) at 488 nm in an Epics XL flow cytometer (Coulter Corporation, Hialeah, FL, USA). For this assay, 5 × 10^4^ B16–F10, 6 × 10^4^ HT29 cells, and 15 × 10^4^ Hep G2 cells were placed in 24-well plates with 1.5 mL of medium and incubated for 24 h. After this time, the cells were treated with the different selected compounds for 72 h at their corresponding IC_50_ concentrations. After treatment, the cells were washed twice with PBS, trypsinized, and resuspended in 1X TBS (10 mM Tris and 150 mM NaCl); thereafter, Vindelov buffer (100 mM Tris, 100 mM NaCl, 10 mg/mL Rnase, and 1 mg/mL PI, at pH 8) was added. Cells were stored on ice and, just before measurement, were stained with 10 μL of 1 mg/mL PI solution. Approximately, 10 × 10^3^ cells were analyzed in each experiment. The experiments were performed three times with two replicates per assay.

### Flow-Cytometry Analysis of the Mitochondrial-Membrane Potential (MMP)

The electrochemical gradient across the mitochondrial membrane was examined by analytical flow cytometry, using dihydrorhodamine (DHR), which in contact with living cells, oxidized to form highly fluorescent rhodamine 123 (Rh123). The fluorescence emitted can be monitored by fluorescence spectroscopy using excitation and emission wavelengths of 500 and 536 nm, respectively. Altogether, 5 × 10^4^ B16–F10, 6 × 10^4^ HT29 cells, and 15 × 10^4^ Hep G2 cells were placed in 24-well plates, and they were incubated for 24 h and treated with the selected compounds (**1**, **4**, **7**, and **10**) for 72 h at their corresponding IC_50_ concentrations. Following treatment, the culture medium was renewed with fresh medium by adding 0.5 mL of DHR, for a final concentration of 5 mg/mL. Cells were incubated for 1 h at 37 °C in an atmosphere of 5% CO_2_ and 95% humidity and subsequently washed and resuspended in PBS. The fluorescence intensity was measured using a FACScan flow cytometer (fluorescence-activated cell sorter). The experiments were performed three times with two replicates per assay.

## Supporting Information Available

Growth inhibitory effects, percentage of apoptosis of three cell lines, flow-cytometry analyses, and ^1^H NMR, ^13^C NMR, and DEPT spectra of compounds (PDF)
