# Supplementary material for: Synthesis and Biological Activity of Triterpene–Coumarin Conjugates
Source: J Nat Prod. 2021 May 6;84(5):1587–97. doi: 10.1021/acs.jnatprod.1c00128 (PMC8476055; doi:10.1021/acs.jnatprod.1c00128)

Supporting Information

Synthesis and Biological Activity of Triterpene-Coumarin Conjugates

Karina Vega-Granados,[†] *Marta Medina-O'Donnell,*[†] *Francisco Rivas,*[*,†] *Fernando J. Reyes-Zurita,*[*,‡] *Antonio Martinez,*[†] *Luis Alvarez de Cienfuegos,* [†] *Jose A. Lupiañez,* [‡] *and Andres Parra*[*,†]

[†] Departamento de Química Orgánica. [‡] Departamento de Bioquímica y Biología Molecular I. Universidad de Granada, E-18071 Granada, Spain.

Table of Contents

Page S3: Table S1. Growth inhibitory effects of MA conjugates 7 and 10 on HPF, IEC-18, and WRL68 non-tumor cells.
Page S4: **Table S2.** Percentage of apoptosis of the three cancer-cell lines, after exposure to the Control and the MA conjugates (**1**, **4**, **7**, and **10**).
Page S5: **Figure S1**. Flow-cytometry analysis of early apoptosis, late apoptosis and necrosis of B16-F10 cancer-cell line after treatment with MA conjugates **1**, **4**, **7**, and **10**.
Page S6: **Figure S2**. Flow-cytometry analysis of early apoptosis, late apoptosis and necrosis of HT29 cancer-cell line after treatment with MA conjugates **1**, **4**, **7**, and **10**.
Page S7: **Figure S3**. Flow-cytometry analysis of early apoptosis, late apoptosis and necrosis of Hep G2 cancer-cell line after treatment with MA conjugates **1**, **4**, **7**, and **10**.
Page S8: **Figure S4**. ^{1}H, ^{13}C NMR spectra and DEPT of compound **1** ($CDCl_3$).
Page S9: **Figure S5**. ^{1}H, ^{13}C NMR spectra and DEPT of compound **2** ($CDCl_3$).
Page S10: **Figure S6**. ^{1}H, ^{13}C NMR spectra and DEPT of compound **3** ($CDCl_3$).
Page S11: **Figure S7**. ^{1}H, ^{13}C NMR spectra and DEPT of compound **4** (CD_3OD).
Page S12: **Figure S8**. ^{1}H, ^{13}C NMR spectra and DEPT of compound **5** (CD_3OD).
Page S13: **Figure S9**. ^{1}H, ^{13}C NMR spectra and DEPT of compound **6** (CD_3OD).
Page S14: **Figure S10**. ^{1}H, ^{13}C NMR spectra and DEPT of compound **7** (CD_3OD).
Page S15: **Figure S11**. ^{1}H, ^{13}C NMR spectra and DEPT of compound **8** (CD_3OD).
Page S16: **Figure S12**. ^{1}H, ^{13}C NMR spectra and DEPT of compound **9** (CD_3OD).
Page S17: **Figure S13**. ^{1}H, ^{13}C NMR spectra and DEPT of compound **10** ($CDCl_3$).
Page S18: **Figure S14**. ^{1}H, ^{13}C NMR spectra and DEPT of compound **12** ($CDCl_3$).

Table S1. Growth inhibitory effects of MA conjugates **7** and **10** on HPF, IEC-18, and WRL68 non-tumor cells.

Compound	HPF (μM)	IEC-18 (μM)	WRL68 (μM)
**7**	38.8 ± 1.3	19.6 ± 0.3	16.3 ± 0.9
**10**	24.5 ± 1.9	7.4 ± 0.7	6.2 ± 0.0

The IC_{50} values (μM) were calculated considering control untreated cells as 100% of viability. Cell-growth inhibition was analyzed by the MTT assay, as described in Experimental section.

Table S2. Percentage of apoptosis of the three cancer-cell lines, after exposure to the Control and the MA conjugates (**1**, **4**, **7**, and **10**).

Cell line	Compound	Viable Cells	Early Apoptosis	Late Apoptosis	Total Apoptosis	Necrosis
B16-F10	Control	91.0 ± 1.1	0.2 ± 0.1	4.2 ± 1.6	4.3 ± 0.9	4.8 ± 2.8
	**1**	30.5 ± 1.9	48.5 ± 2.8	19.4 ± 4.2	67.9 ± 1.5	1.7 ± 0.4
	**4**	14.0 ± 3.9	74.9 ± 2.6	10.5 ± 1.4	85.4 ± 3.0	0.7 ± 0.0
	**7**	39.3 ± 3.5	39.0 ± 4.1	18.6 ± 0.1	57.6 ± 3.2	3.1 ± 0.6
	**10**	53.4 ± 2.9	18.1 ± 3.0	22.9 ± 3.8	41.0 ± 1.7	5.7 ± 0.8
HT29	Control	89.5 ± 4.3	1.2 ± 2.4	7.7 ± 2.3	8.8 ± 1.4	1.7 ± 0.3
	**1**	56.3 ± 1.3	10.8 ± 0.6	29.2 ± 0.5	40.0 ± 0.5	3.7 ± 0.9
	**4**	41.4 ± 3.8	32.0 ± 2.3	20.5 ± 3.0	52.4 ± 3.0	6.2 ± 0.9
	**7**	52.9 ± 2.0	16.2 ± 3.6	29.4 ± 1.6	45.6 ± 2.0	1.5 ± 0.4
	**10**	35.7 ± 2.3	23.8 ± 4.2	34.1 ± 4.9	57.9 ± 1.6	6.5 ± 0.0
Hep G2	Control	93.0 ± 0.8	2.4 ± 0.5	1.3 ± 2.3	5.7 ± 0.2	1.3 ± 0.3
	**1**	28.8 ± 2.8	50.1 ± 4.5	19.4 ± 4.8	69.5 ± 2.3	1.8 ± 0.4
	**4**	29.7 ± 1.3	48.5 ± 3.8	20.1 ± 5.3	68.5 ± 1.6	1.9 ± 0.2
	**7**	21.9 ± 1.3	57.4 ± 4.3	18.6 ± 3.2	75.9 ± 2.0	2.2 ± 0.6
	**10**	55.0 ± 1.4	19.2 ± 4.9	21.6 ± 3.2	40.8 ± 3.2	4.2 ± 1.1

Flow-cytometry analysis of Annexin V-FITC staining and PI accumulation (apoptosis) after exposure of the three cancer-cell lines to the Control and MA conjugates (**1**, **4**, **7**, and **10**) for 72 h. Cell lines were treated at concentrations equal to their corresponding IC_{50} values. Values are expressed as means ± S.E.M. of at least two duplicate experiments.

Figure S1. Flow-cytometry analysis of early apoptosis, late apoptosis and necrosis of B16-F10 cancer-cell line after treatment with MA conjugates **1**, **4**, **7**, and **10**.

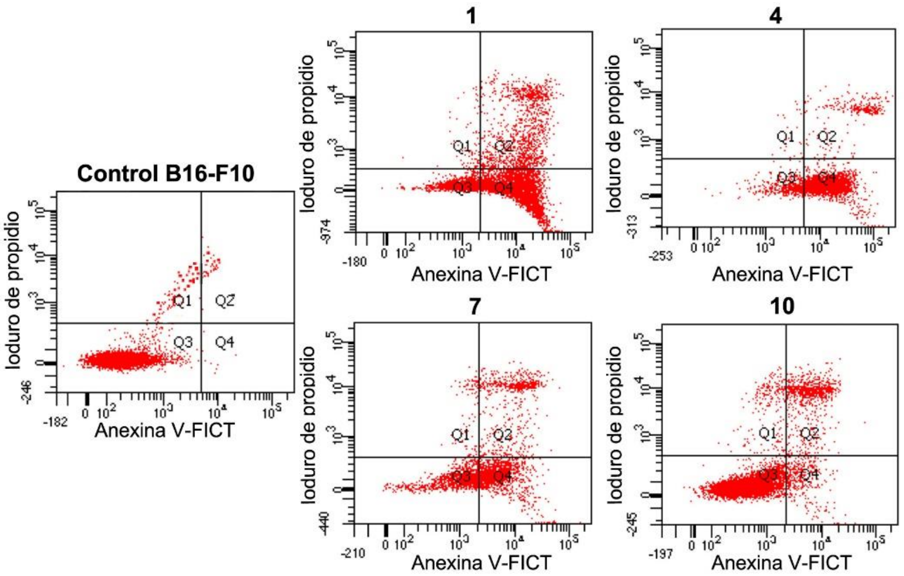

Figure S2. Flow-cytometry analysis of early apoptosis, late apoptosis and necrosis of HT29 cancer-cell line after treatment with MA conjugates **1**, **4**, **7**, and **10**.

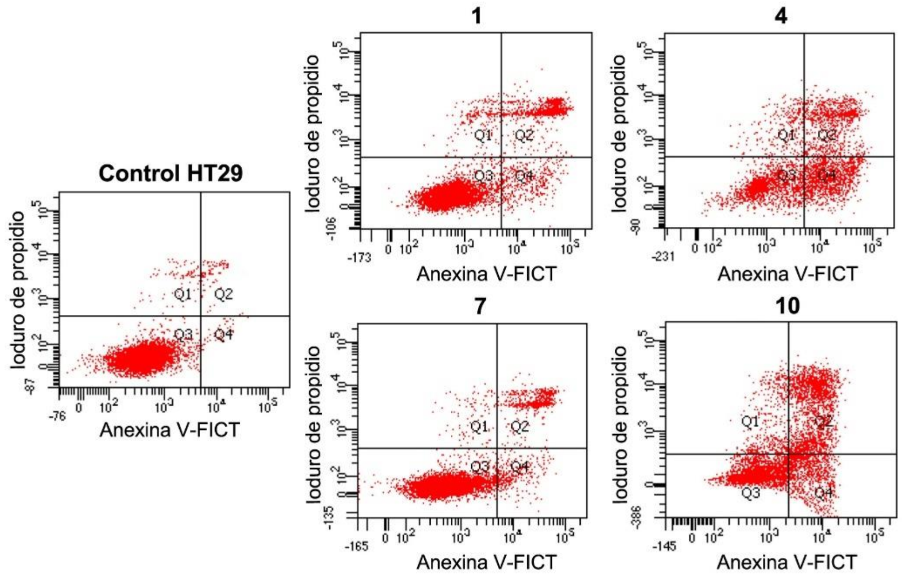

Figure S3. Flow-cytometry analysis of early apoptosis, late apoptosis and necrosis of Hep G2 cancer-cell line after treatment with MA conjugates **1**, **4**, **7**, and **10**.

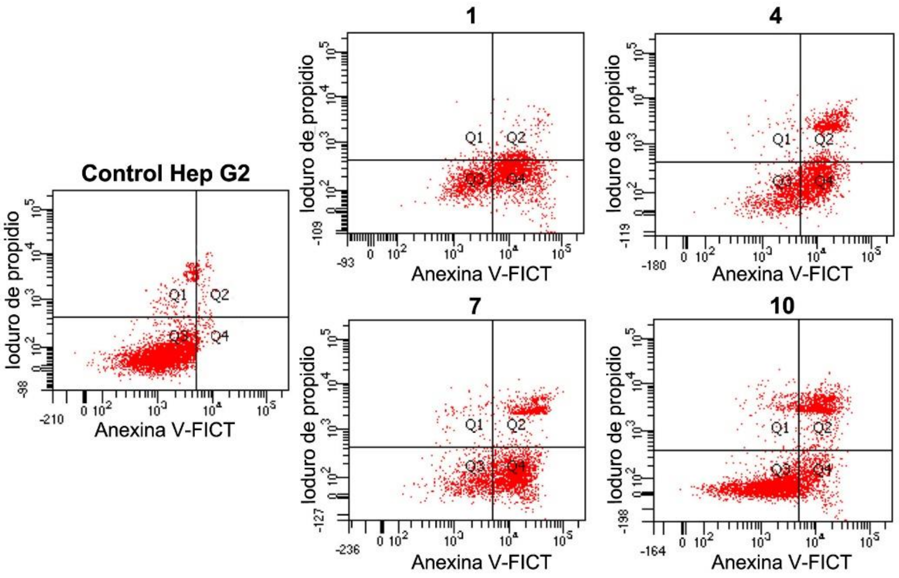

Figure S4. ^{1}H, ^{13}C NMR spectra and DEPT of compound **1** ($CDCl_3$).

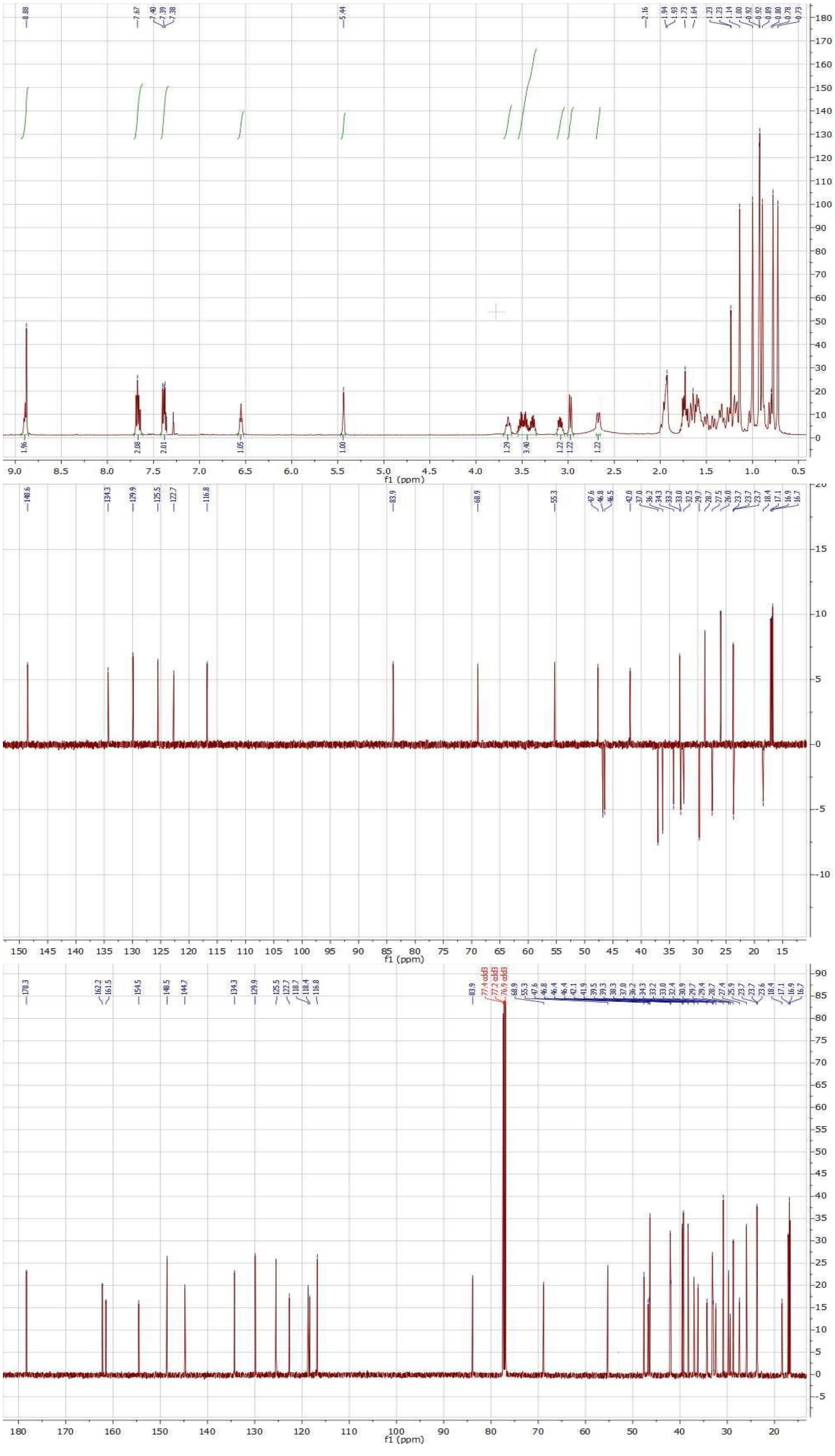

Figure S5. ^{1}H, ^{13}C NMR spectra and DEPT of compound **2** ($CDCl_3$).

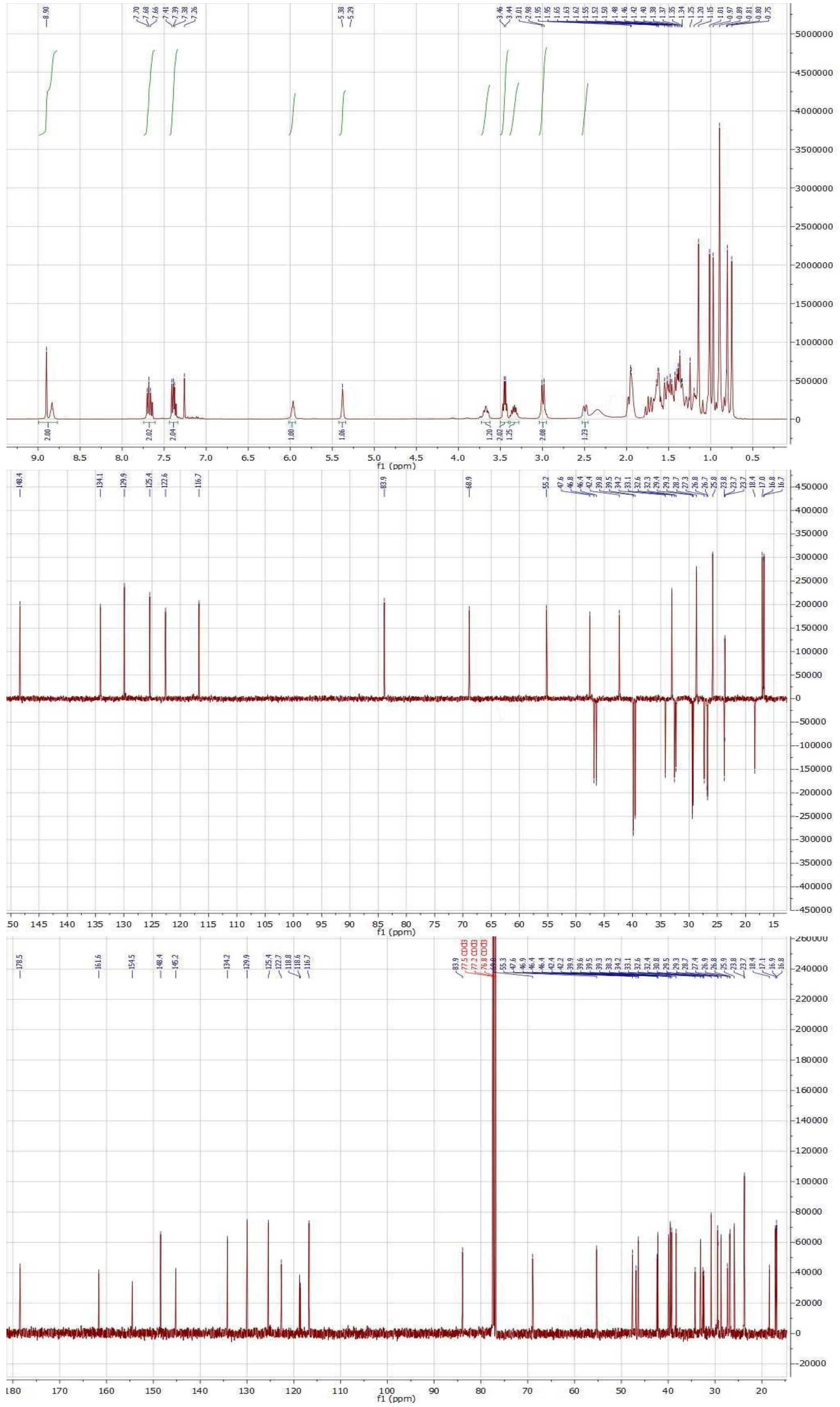

Figure S6. ^{1}H, ^{13}C NMR spectra and DEPT of compound **3** ($CDCl_3$).

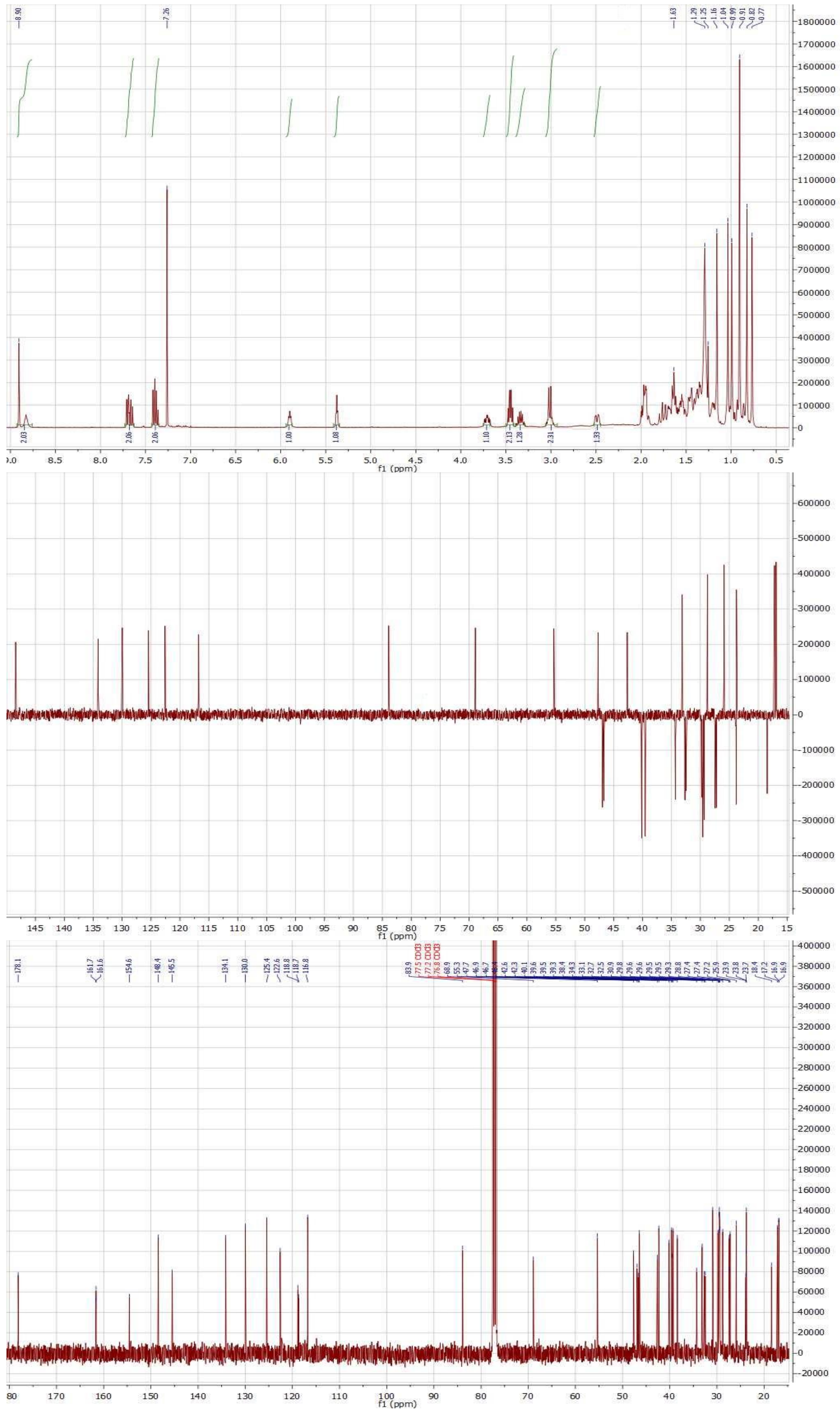

Figure S7. ^{1}H, ^{13}C NMR spectra and DEPT of compound **4** (CD_3OD).

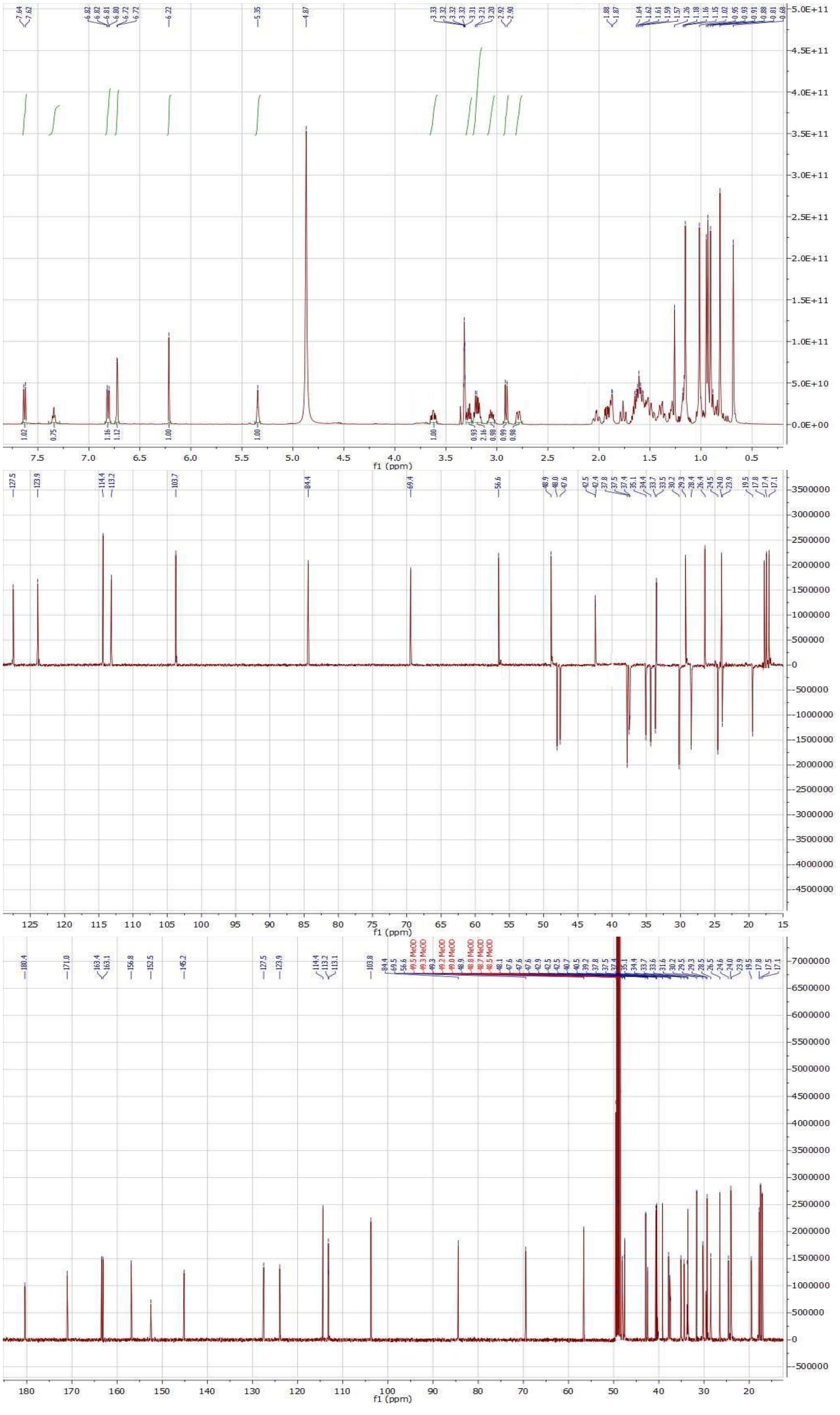

Figure S8. ^{1}H, ^{13}C NMR spectra and DEPT of compound **5** (CD_3OD).

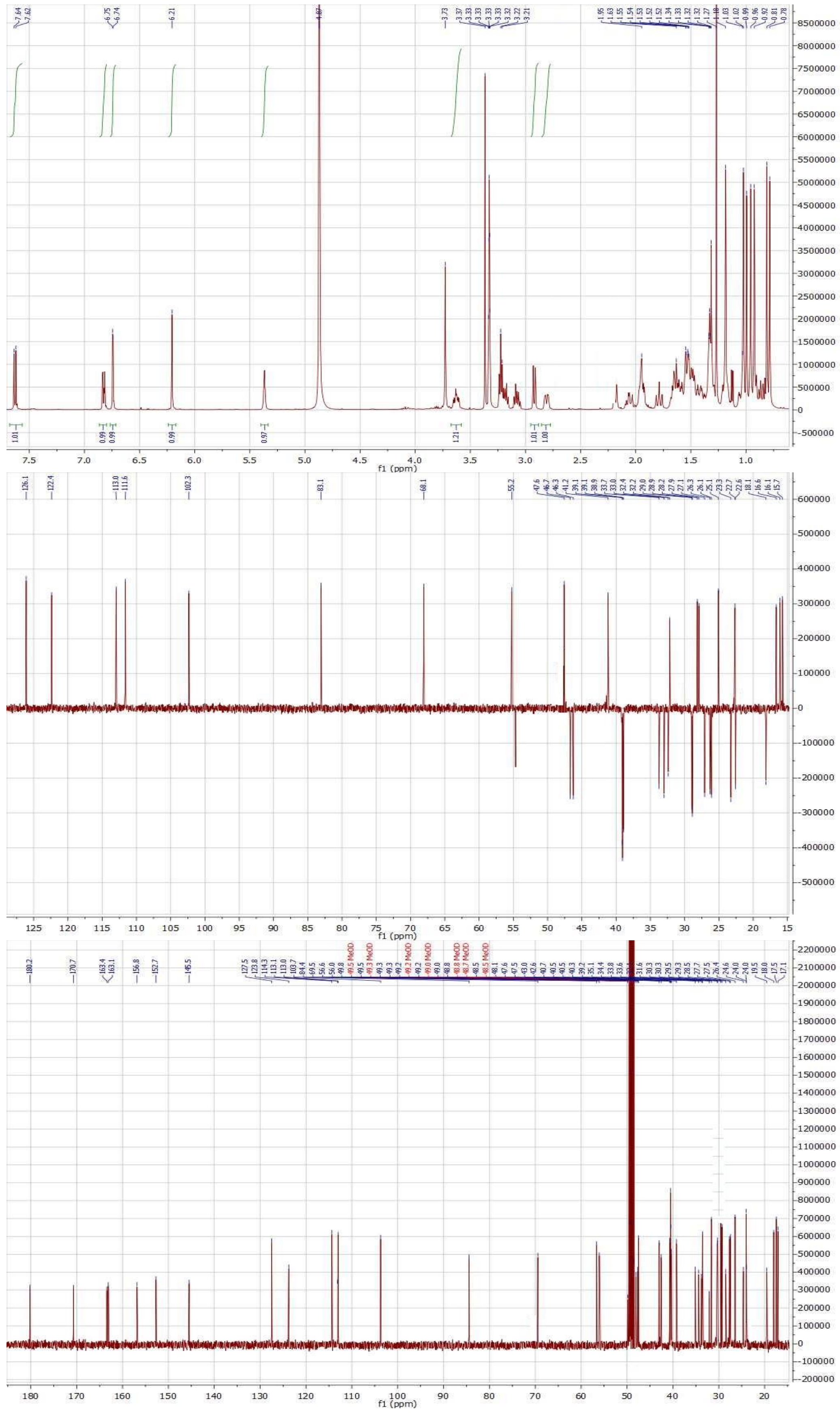

Figure S9. ^{1}H, ^{13}C NMR spectra and DEPT of compound **6** (CD_3OD).

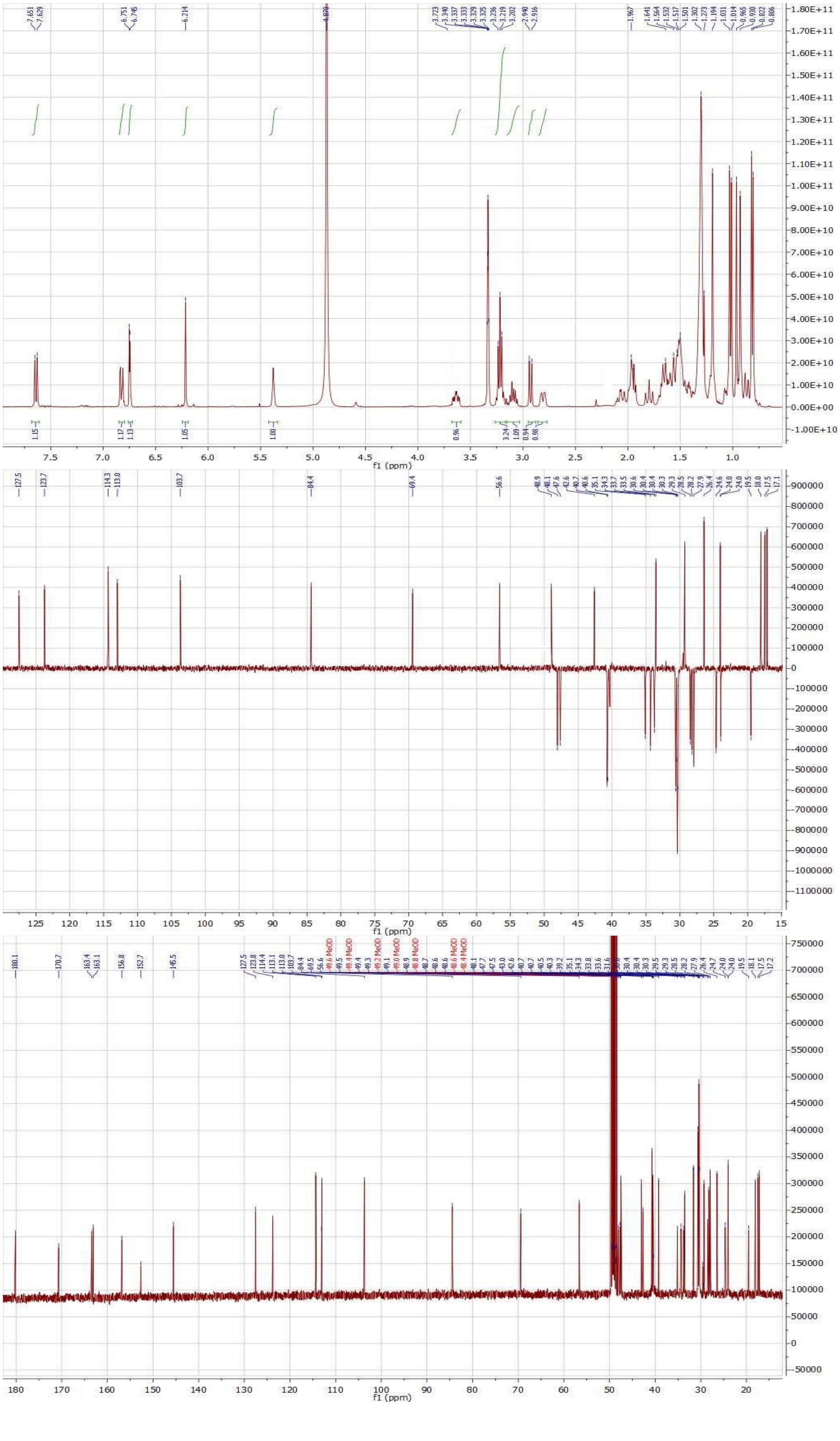

Figure S10. ^{1}H, ^{13}C NMR spectra and DEPT of compound **7** (CD_3OD).

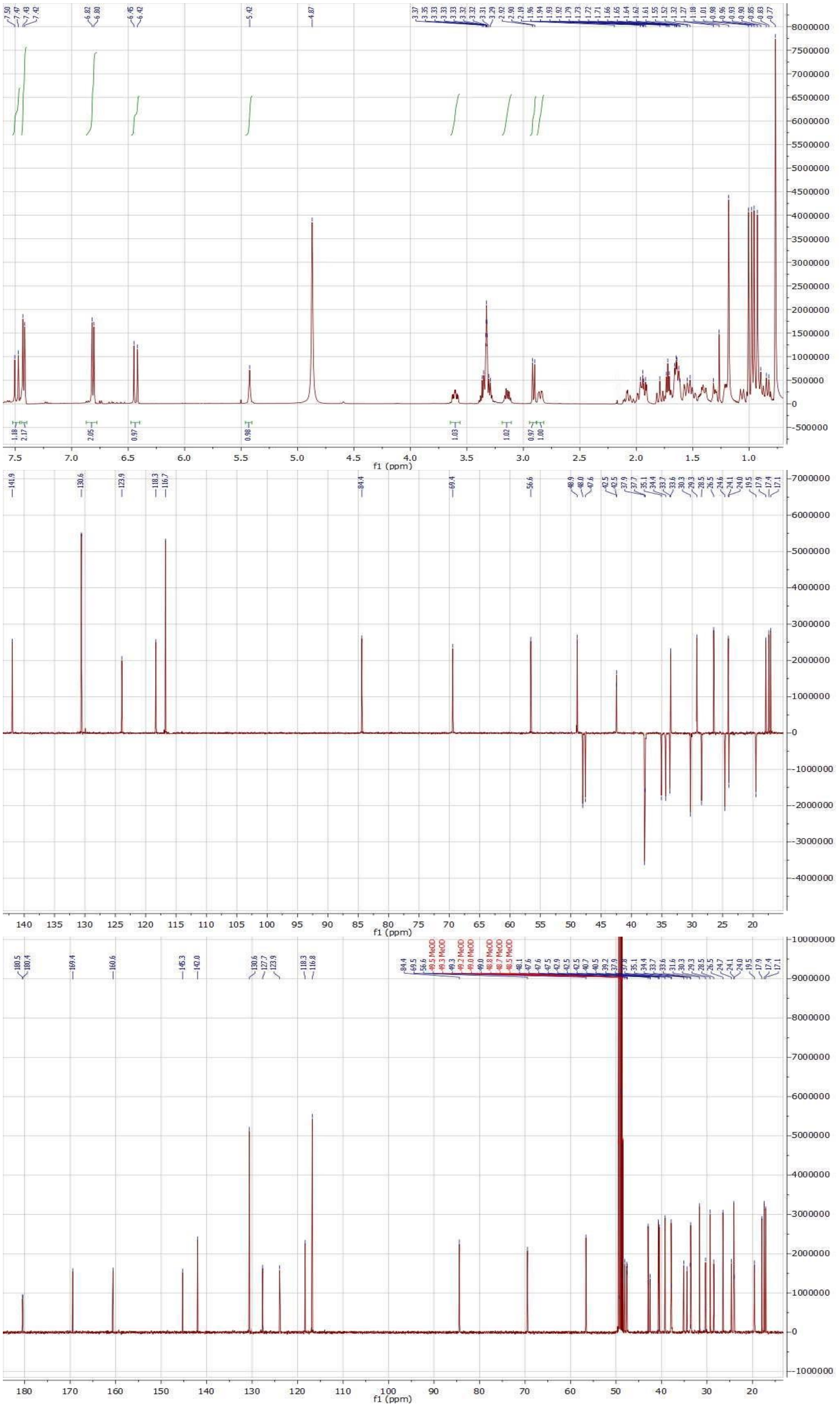

Figure S11. ^{1}H, ^{13}C NMR spectra and DEPT of compound **8** (CD_3OD).

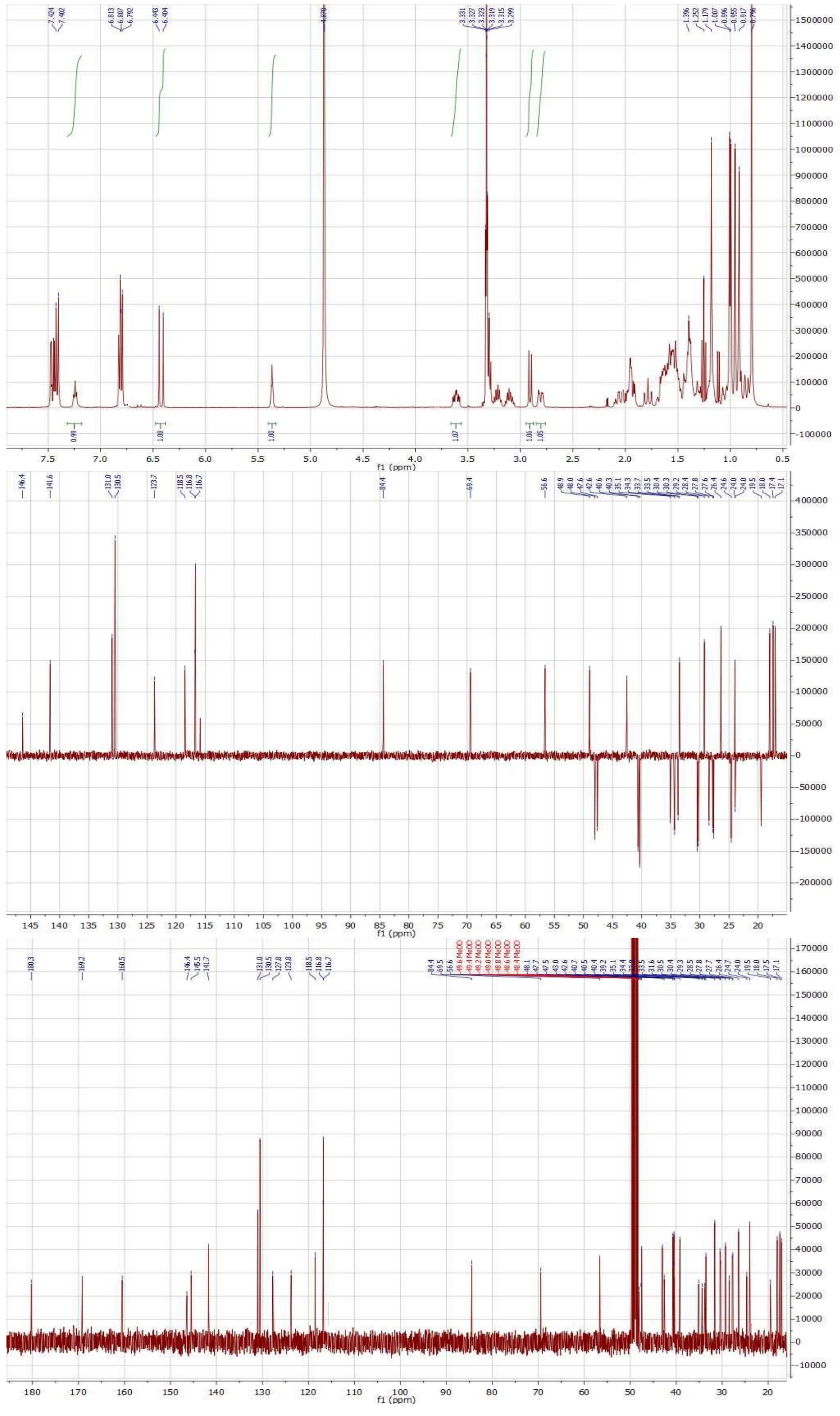

Figure S12. ^{1}H, ^{13}C NMR spectra and DEPT of compound **9** (CD_3OD).

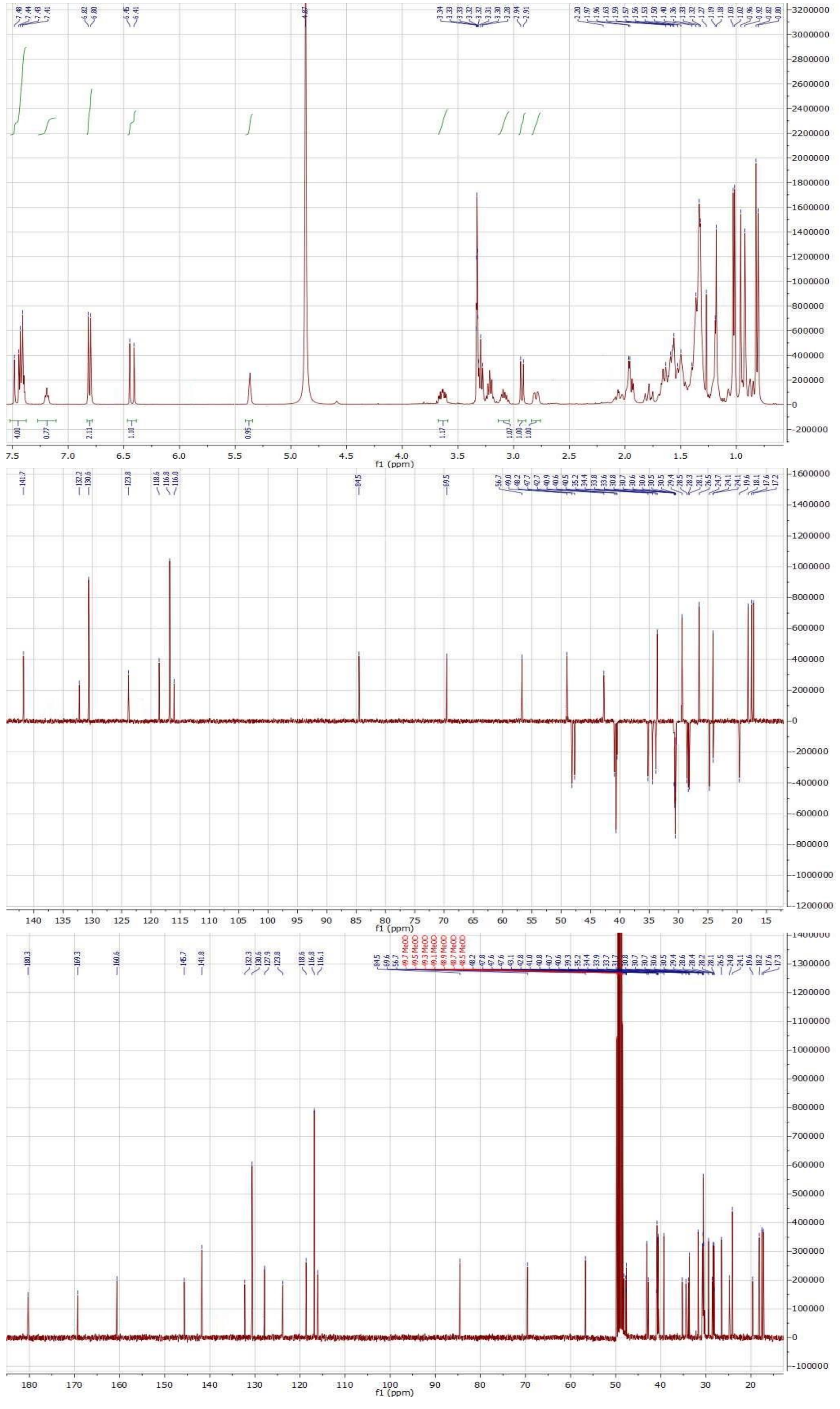

Figure S13. ^{1}H, ^{13}C NMR spectra and DEPT of compound **10** ($CDCl_3$).

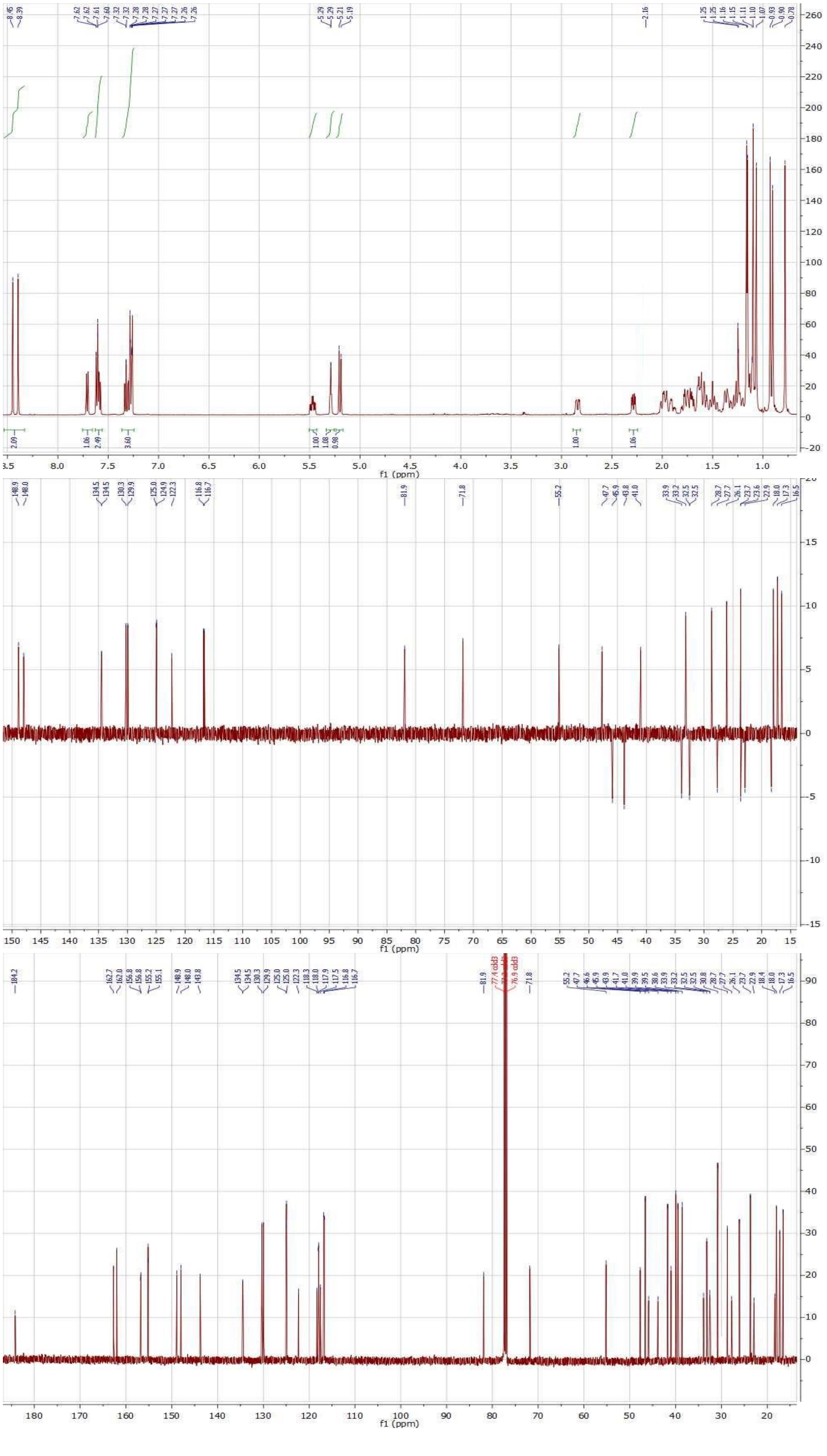

Figure S14. ^{1}H, ^{13}C NMR spectra and DEPT of compound **12** ($CDCl_3$).

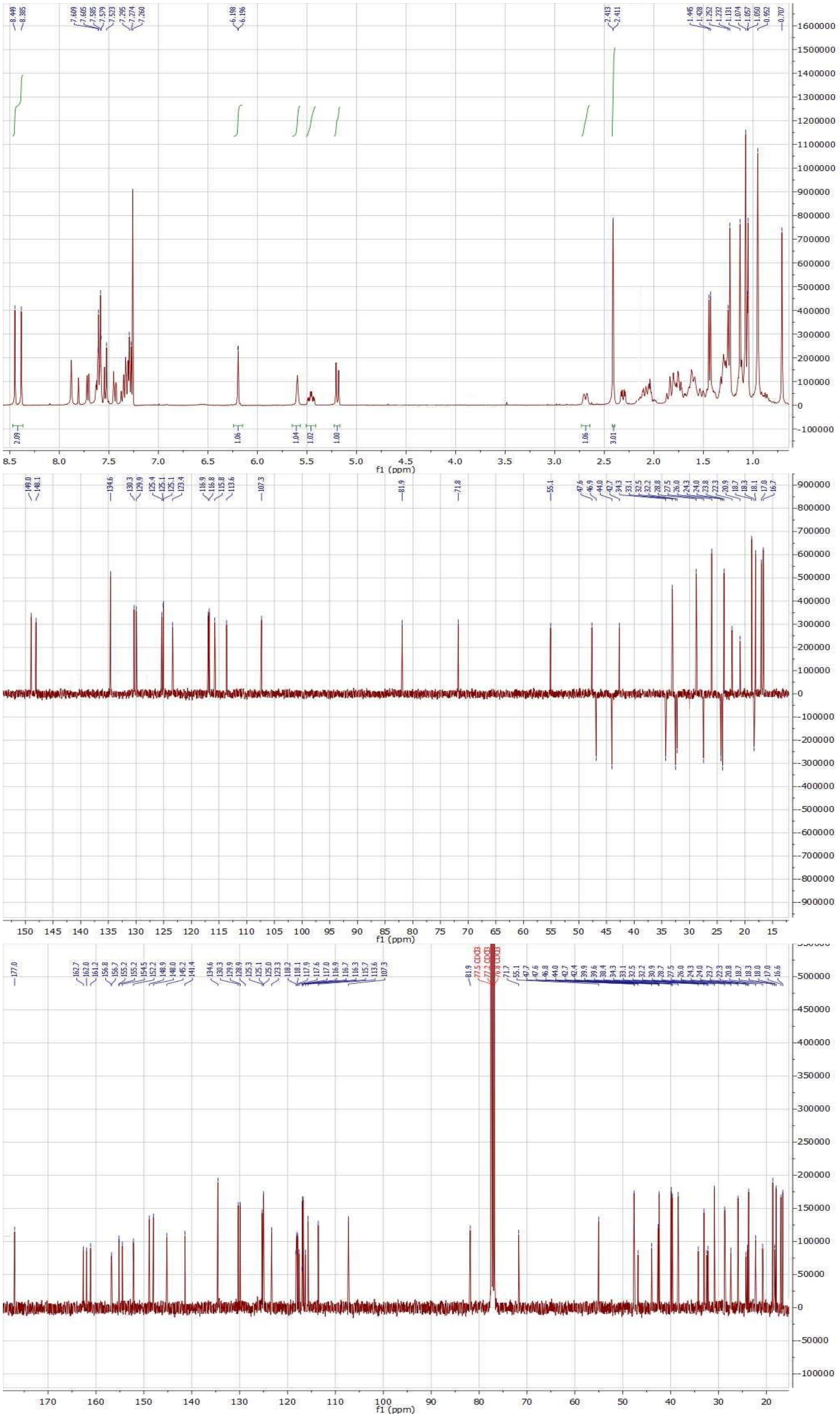

Supplement: Supplementary file 1 — np1c00128_si_001.pdf [file np1c00128_si_001.pdf]
